# Natural and sexual selection and functional roles influence colouration but not the amount of variation in butterfly wing colour patterns

**DOI:** 10.1186/s12862-024-02346-8

**Published:** 2025-01-17

**Authors:** Bhavya Dharmaraaj, Krushnamegh Kunte

**Affiliations:** National Centre for Biological Sciences, Tata Institute of Fundamental Research, GKVK Campus, Bellary Road, Bengaluru, 560065 India

**Keywords:** Phenotypic variation, Sexual dimorphism, Spectral properties, Strength of selection, Trait variance

## Abstract

**Background:**

Trait variation is shaped by functional roles of traits and the strength and direction of selection acting on the traits. We hypothesized that in butterflies, sexually selected colouration is more variable owing to condition-dependent nature and directional selection on sexual ornaments, whereas naturally selected colouration may be less variable because of stabilising selection. We measured reflectance spectra, and extracted colour parameters, to compare the amount of variation in sexually versus naturally selected colour patches across wing surfaces and sexes of 20 butterfly species across 4 families (Nymphalidae, Papilionidae, Pieridae, Lycaenidae).

**Results:**

We found that: (a) males had more conspicuous, i.e., brighter and more saturated colour patches compared with females (as expected of sexually selected traits but not necessarily of naturally selected traits), and (b) dorsal surfaces in both sexes had more conspicuous sexual ornaments as well as protective (aposematic/mimetic) colour patches on darker wing backgrounds, compared with ventral surfaces. However, colour patches did not differ in the amount of variation either in selection (ecological/sexual functions), sex or wing surface-specific manner.

**Conclusions:**

These findings show that functional roles and selection influence colour parameters but not the amount of variation in butterfly wing colour patterns.

**Supplementary Information:**

The online version contains supplementary material available at 10.1186/s12862-024-02346-8.

## Background

Phenotypic variation and the underlying genetic variation are the primary raw material for selection to act on, resulting in adaptation, diversification and speciation. Consequently, understanding how natural and sexual selection shape variation at both genetic [1] and phenotypic [2, 3] levels is of broad interest to evolutionary biologists. Studies on mean phenotypic variation within and across populations have provided information on the identity and strength of various selective pressures affecting mean trait variation [4]. For example, oscillations in cycles of drought and rainfall that altered the available food resources resulted in temporal alterations in trait optima for body size and measures of beak morphology in a species of Darwin’s finch, *Geospiza fortis* [5]. Similarly, changes in trait optima of sexual signals have been attributed to changing mate preference [6] or signal exploitation by predators and parasites [7, 8]. While these examples highlight how means of traits are subject to change, or how traits may be lost and gained, the strength and direction of selection can affect the variability of traits to different extents, and variability can in turn influence future adaptation.

**Fig. 1:**
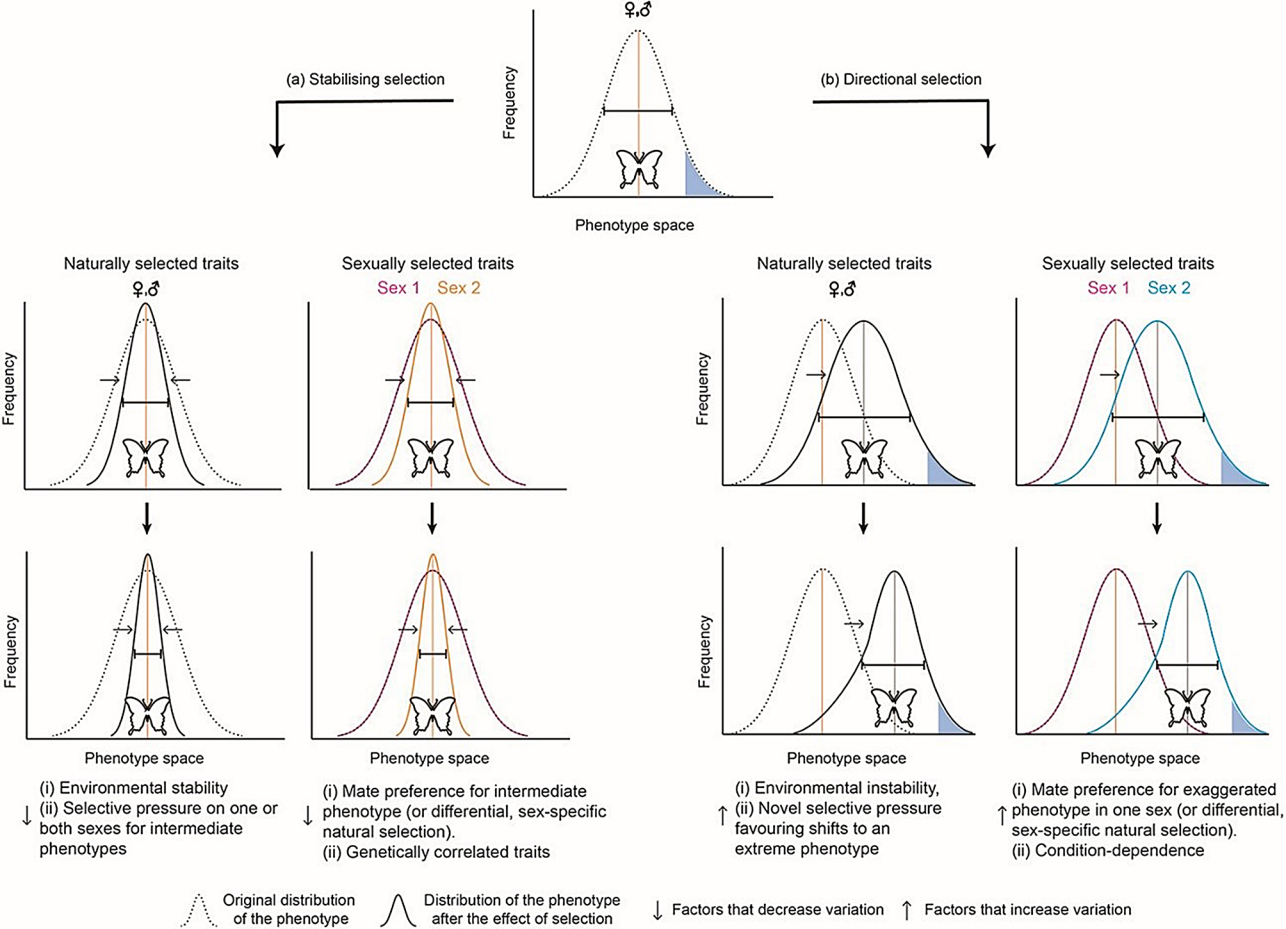
A graphic summary of hypotheses regarding how functional roles and selection pressures may influence trait variation. Thin arrows represent direction of selection and thick arrows represent direction of evolution for each column. Flat-headed lines indicate the relative spread of trait variation. The orange line in **(a)** represents trait optimum. The blue-shaded area in **(b)** represents the direction in which selection shifts the trait distribution. Stabilising selection on naturally or sexually selected traits **(a)** acting on one or both sexes may decrease trait variation while directional selection on naturally or sexually selected traits **(b)** may increase trait variation. In the panels for sexually selected traits, the effect of selection on trait variation has been shown separately to highlight how variation between the sexes may respond differentially. Additionally, natural selection can also affect sexes differently, potentially resulting in distinct patterns of variation between males and females, similar to what is shown for sexually selected traits. However, even in such cases, the variation in naturally selected traits is generally expected to be less pronounced than in sexually selected traits or traits not under selection

Selection acting on a trait affects the amount of variation such that: (a) stabilising selection maintains the mean trait value but reduces variation in a population by disfavouring individuals at the tails of the distribution (Fig. 1a) [9, 10] and (b) directional selection displaces the trait optimum towards one side of the distribution, potentially leading to an initial increase in variation during the population’s shift towards the new optimum [9, 10]. Traits under directional selection may eventually experience reduction in variation when the new optimum is reached, albeit to a lesser extent than long-term stabilising selection [9] (Fig. 1b). Moreover, trait values and variation may remain dynamic and not achieve a new, stable optimum distribution if directional selection is persistent, e.g., for sexual ornaments under runaway-type sexual selection [11, 12]. How the variability of the trait increases or decreases during the lifespan of these selective processes, and whether the sexes are affected differentially, are poorly understood. The strength of natural (or viability) selection and sexual selection also affect the evolutionary trajectories of traits where sexual selection may be stronger than natural selection and can push trait optima further along the naturally selected limit, especially in males [9, 13, 14]. Sexual signals can evolve to exaggerated levels through the correlated evolution of female preference for attractive male traits (sexy-sons) or when the signals provide information on the quality of the signaller (good genes) [15].

Sexually selected traits are often condition dependent with greater genetic variance [16]. Empirical studies highlighting cases of condition dependence and variable nature of sexual traits [17] include sexual size dimorphism in a neriid fly [18] and eye span in male stalk-eyed flies [19, 20]. In contrast, naturally selected traits, such as colours and colour patterns used in Batesian mimicry, may be more [21, 22] or less [23] variable depending on the abundance and distribution of the Batesian models [21–23]. However, at ecological timescales, these naturally selected traits should be under variation-reducing stabilising selection since closer mimetic resemblance usually confers greater fitness advantage [24, 25]. Therefore, how different functions and selective pressures affect phenotypic variation remains an area of significant interest.

Animal colour signals are storehouses of variation upon which diverse selection pressures can act, affecting phenotypic variation, diversification, and speciation [26]. Understanding the nature and variability of sexually and naturally selected colour patterns is key. Examining condition-dependent variation of sexual colouration versus non-sexual colouration has received considerable attention in diverse taxa such as birds [27–30], fishes [31], and jumping spiders [32].

The importance of wing colouration in visual signalling is exemplified in butterflies. Butterfly wing colours perform a number of signalling functions ranging from predator avoidance [33–37] to mate-signalling [38–41]. Wing colouration also responds to physiological processes such as thermoregulation [42, 43]. Thus, butterfly wing colours are diverse and subject to both natural selection to facilitate predator avoidance and thermoregulation, and sexual selection, to facilitate mate-choice through courtship where different colour patches may be under stabilising or directional selection.

Variation in butterfly colour patterns is subject to the nature of colour production. Butterfly wing colouration may be pigmentary or structural [44]. Structural colouration, often used in sexual ornaments, is expected to be more visually variable than pigment based colouration because it tends to be condition dependent (e.g., on the angle of light in iridescent colour patches) and it may signal higher male quality [45], resulting in more matings [46, 47]. Sexually selected pigmentary colouration can also differ between males and females in amount of pigments deposited on the scales [48, 49]. But, unlike structural colours, the appearance of pigment patches is not affected by the angle of light and viewing.

However, predominantly naturally selected colouration can also be variable. Factors such as resource limitation and environmental stress during larval stages affects some pigmentary warning colouration in aposematic species [50] as well as melanic thermoregulatory colouration in adult butterflies [51]. Colour patterns may thus vary across sex and wing surfaces in relation to these ecological, developmental and sexual functions and sex-specific selection pressures. Indeed, female colouration is more variable than male colouration [52], in its naturally selected roles such as melanic thermoregulation and Batesian mimicry [42, 53], where females are often polymorphic, or with respect to life-history strategies such as dispersal and migration [54, 55]. Therefore, these sex-specific and patch-function-dependent contrasts in variability make butterflies an excellent model system to study how different selective regimes shape colour pattern variation in relation to functional roles of those colour patches.

A major gap in this area is that most previous studies have addressed the question of whether traits such as wing colour patterns respond to different selective regimes, and whether males and females respond differentially to selective regimes. Most individual studies have also usually focused on patterns of colour variation in single species, and not as a broader generalization. Moreover, whether variation in colour patterns itself responds in relation to sex, wing surface and ecological/sexual roles of the colour patches, has rarely been investigated. It has been phylogenetically demonstrated, in *Bicyclus* butterflies, how evolutionary rates of wing patterns differ in sex- and surface-specific manners to accommodate contrasting selective pressures resulting in the observed variation [56]. However, addressing this gap from a proximate viewpoint is important as well because the amount of variation observed in a species may reflect the evolutionary history of sex and surface-specific responses to selection. More importantly, sex-specific and population-level ability to respond to future selection pressures is determined by available variation. This aspect is critical in light of the rapidly changing environment and habitat alternations in globally human-dominated landscapes in which most populations have to now adapt to survive. In order to begin to address this gap in understanding colour variation, we quantified spectral properties of colour patches using a spectrophotometer and asked: (a) whether butterfly wing colour patterns vary in a sex and wing surface-specific (dorsal vs. ventral) manner in a set of species that displayed a variety of colour patterns produced in a structural versus pigmentary manner, and that were subjected to natural versus sexual selection, and (b) whether the colour patches showed consistent patterns regarding the amount of variation in response to sex, wing surface and type of selection pressures. Based on the above background, we specifically tested the following hypotheses regarding the nature of differences and the extent of variation: (a) male wing colour patches are more conspicuous (i.e., with spectrally brighter or more saturated colours) than female wing colour patches, (b) dorsal wing colour patches are more conspicuous than ventral wing colour patches (dorsal surfaces are usually displayed during active courtship, which is usually by the males, while ventral surfaces tend to be used in anti-predator strategies [40, 57–59]), (c) colour patches of females show greater degree of variation than that of colour patches of males, (d) sexually selected colour patches show greater variation than naturally selected colour patches, (e) structural colour patches show greater variation than pigmentary colour patches.

## Materials and methods

### Butterfly specimens and colour patch functions

We measured wing colours of butterfly specimens deposited in the Biodiversity Lab Research Collections at NCBS (http://biodiversitycollections.in). We selected species that had 3–10 specimens of each sex of four butterfly families (Nymphalidae, Papilionidae, Pieridae, Lycaenidae) displaying aposematic, mimetic, cryptic, UV-reflective, iridescent, or fluorescent patches (Table S1 for species used and Table S2 for reflectance spectra). These samples sizes, though small, have been shown to be useful in documenting within and between species colour differences accurately [60]. We classified these colours based on studies which have directly investigated functional roles of colour patterns such as in aposematic, mimetic and thermoregulatory species, and certain sexual ornaments. For cases lacking direct evidence, we inferred functions from studies on other species with similar phenotypes, such as those exhibiting sexual or iridescent coloration (Table S1 and Fig. S2 provides further details with references).As the entire pattern would contribute to the signalling functions, we grouped all wing colour patches under the regime of natural selection if they were part of aposematic or mimetic signals, or if they aided in camouflage or thermoregulation [42, 53, 59, 61–65]. Whereas we classified only UV-reflective, iridescent, and fluorescent colour patches as sexually selected colours or sexual signalling colours based on existing knowledge about the sexual role of such colour patches [39, 40, 64, 66–71]. Although colour patterns may sometimes be used for multiple functions across selective regimes [72, 73], we proceeded under the assumption that some functions were more widespread, relevant, or supported by previous studies than the others. For example, we considered wing patterns of aposematic butterflies to be primarily predator-driven (naturally selected) though aposematic signals might function as sexual signals as well, which might sometimes evolve secondarily [74]. Due to this method of classification, most species had colour patches of only one well-supported function. Multiple functions were not considered unless there were surface or sex-specific dimorphisms for example, *Hypolimnas bolina* where females are mimetic [53, 59, 61], and males are nonmimetic with a bright iridescent white sexual ornament [40] (detailed in Table S1). This is a conservative functional classification but ensured relevant functions were included. This also resulted in several patches, especially in sexually selected species, to be considered as non-specific as we did not have direct or indirect evidence to define functions for these other patches.

### Reflectance spectra measurements

We measured reflectance spectra from all visually distinct wing patches of both sexes across both wing surfaces using Ocean Optics^®^ Jaz spectrometer with a pulsed Xenon lamp (PX-1 lamp) as the light source. We used two optic fibres, fitted with collimating lenses, to illuminate the wing and collect reflectance. We placed the illuminating probe at 90° and the collecting fibre at 45° to the wing surface. The beam of incident light had a diameter of ~ 2 mm. We took measurements with respect to a Spectralon^®^ reflectance standard which reflects > 96% of incident light. We excluded ambient light by enclosing the set up in a cardboard box covered by black felt cloth. We took 2–4 measurements per patch depending on its extent on the wing. We averaged these measurements and used the averaged spectra from 300 to 700 nm to extract colour parameters. We plotted spectra across males and females and dorsoventral wing axis (Supplementary Fig. S1) and obtained the spectral colour parameters using the R package ‘pavo’ [75].

### Spectral parameters

We extracted parameters for hue, saturation, and brightness for all colour patches on both wing surfaces and sexes. As most of the spectra do not have a wavelength of peak reflectance, we used the wavelength at the midpoint of the reflectance spectrum as a measure for hue (H3). We used an overall estimate of chroma to account for saturation of all the colours measured as the difference between maximum and minimum reflectance with respect to the average brightness of the spectrum (S8). We calculated brightness as total reflectance across the whole spectrum (B1). We also extracted segment specific chroma for yellow, red-orange, and blue colour patches (S1) and compared wavelength of peak reflectance (H1) for blues. These notations used are as per [75].

### Statistical analyses

We carried out all tests in R [76]. We tested for normality using Shapiro-Wilk tests. We first tested for differences in brightness, hue, and saturation across sex and surface (dorsal and ventral) within each species separately for each colour using generalized linear models (GLMs) and the ‘gaussian’ family and ‘identity’ link function. We used T-tests and Wilcoxon rank-sum tests for two-sample comparisons between males and females. Following the GLMs, we used the ‘emmeans’ package [77] to obtain contrasts for pairwise differences between the combinations of sex and surface. Next, to determine variability of colour patches, we calculated the coefficients of variation (CV) for the selected colour parameters: B1, H3, and S8, separately across sex and surface, a higher value indicating greater variation in a dataset. We paired the CVs across sex and surface to identify the instances where males had higher values and used one-tailed binomial tests to check if the cases where males had higher values than females were significantly higher across the dorsoventral axis within given selective regimes and within a specific colour. In addition, we also checked for normality of the CVs in a sex and surface-specific manner using the Shapiro-Wilk test and used two-tailed Wilcoxon signed-rank exact test on this paired data to verify differences in coefficients of variation. We used Wilcoxon rank-sum exact tests for dorsoventral comparisons of CVs within a selective regime and colour. Further, to test if CVs varied by selective regimes regardless of sex or surface, we compared them across ecologically relevant function of colour patches using a Kruskal-Wallis test. We used the ‘rstatix’ package [78] to calculate effect sizes for each of the CV comparisons; ‘r’ for two-sample tests and Eta squared (η^2^) for Kruskal-Wallis tests. We also kept colours that did not have a clear association to any specific function to compare variation across these non-specific and functional colour patches.

## Results

### When sexual differences existed, males had brighter and more saturated colour patches than females

**Fig. 2:**
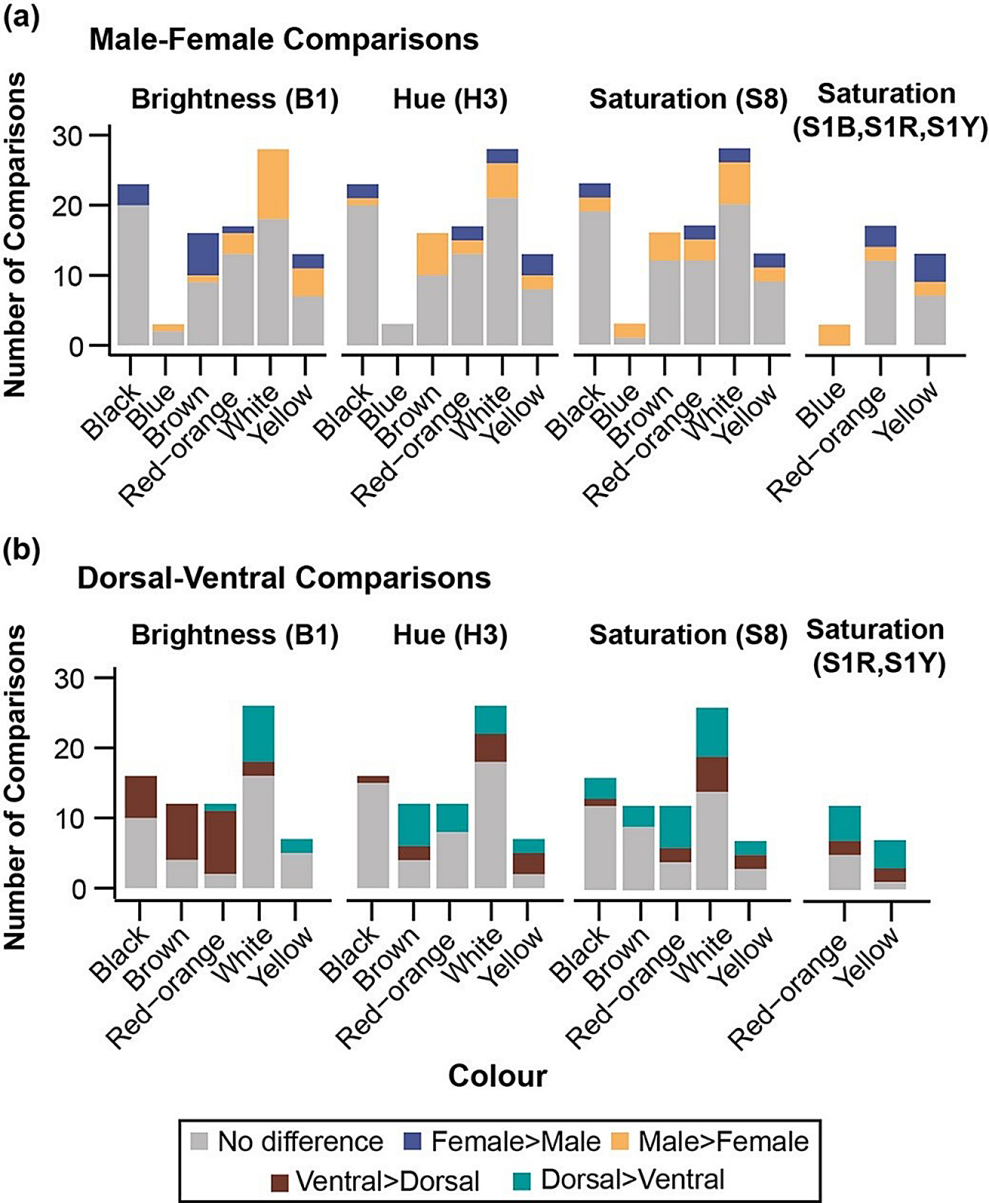
A summary of sex **(a)** and wing surface-wise **(b)** comparisons of the spectral parameters of different colours showing total number of comparisons, comparisons that were not variable, and comparisons that were variable along with the direction of variation

We compared male-female differences in the colour parameters separately for black, brown, white, red-orange, yellow, and blue colour patches, as follows: (a) Variation in brightness: Values of colour parameters did not vary significantly between the sexes in most comparisons (70% of the comparisons). In patches that differed significantly between the sexes (30% of the comparisons), males had darker black and brown wing colour backgrounds than females. However, males had brighter white, red-orange, yellow and blue colour patches that presumably performed functional roles as described in Table S1. (b) Variation in hue: Similarly, only a small proportion of species showed sexual differences in hue (25% of the patches compared), where males had higher values for hue in brown and white patches while values for hue were higher in females for black and yellow patches. (c) Variation in saturation: Likewise, only a small proportion of species showed sexual differences in saturation (29% of the patches compared), where males had more saturated brown, white, red-orange, and blue patches while black and yellow colour patches showed the opposite pattern of being more saturated in females (Figs. 2 and S2a–f, Supplementary Tables S3–S4). Comparisons for wing-surface are provided in the next section.

### Dorsal wing surfaces were more saturated with brighter white and yellow patches than ventral wing surfaces

Using the same colour classification (but excluding blue as there were no sufficiently large blue patches on ventral wing surfaces in the species measured), colour patches differed across the two wing surfaces within each species as follows: (a) Variation in brightness: 49% of the patches differed in brightness between dorsal and ventral surfaces. Within these differences, dorsal surfaces had darker black and brown colour patches, and brighter white and yellow patches than ventral surfaces which had brighter red-orange patches. (b) Variation in hue: Approximately 35% of the patches varied in hue. Red-orange and brown had higher values of hue on the dorsal surface whereas yellow and black had higher values on the ventral surface, with white having equal proportions of higher dorsal and ventral values. (c) Variation in saturation: Saturation differed in approximately 42% of the patches along the dorsoventral axis. All the colours were more saturated on the dorsal surface except for yellow (for all results in this subsection, see Figs. 2 and S2a–e, and Tables S3–S4).

### Functional roles, sexes and wing surfaces did not affect the amount of trait variation

#### Variation in relation to the sexes

**Fig. 3:**
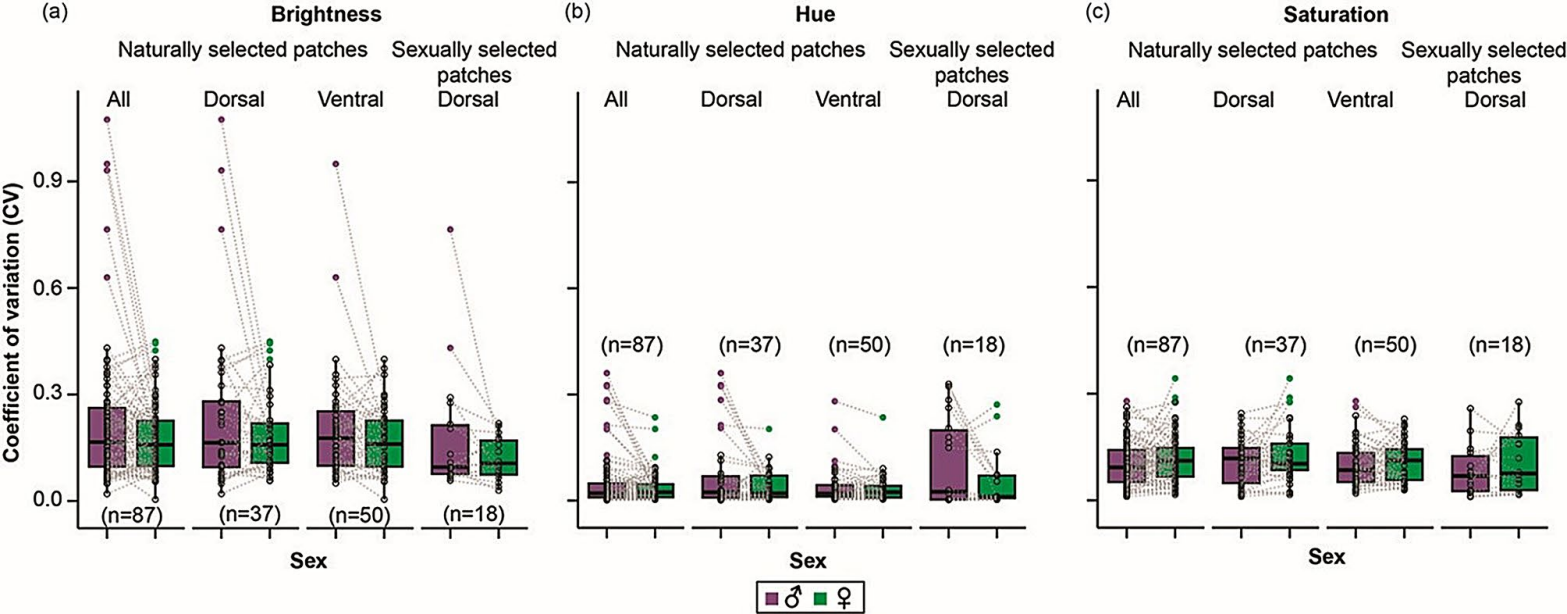
Boxplots of coefficients of variation for brightness **(a)**, hue **(b)**, and saturation **(c)** for naturally and sexually selected colour patches between males and females

We compared coefficients of variation for brightness (B1), hue (H3), and saturation (S8) between males and females for all naturally and sexually selected colour patches. Male wing colour patches were not more variable than female wing colour patches across surface or selective regime for any of the colour parameters (One-tailed Binomial test: **B1**: all naturally selected patches: 44 of 87, *p =* 0.5, naturally selected dorsal patches: 19 of 37, *p =* 0.5, naturally selected ventral patches: 25 of 50, *p =* 0.56, sexually selected patches: 11 of 18, *p =* 0.24. **H3**: all naturally selected patches: 48 of 87, *p =* 0.19, dorsal naturally selected patches: 21 of 37, *p =* 0.26, ventral naturally selected patches: 27 of 50, *p =* 0.36, sexually selected patches: 9 of 18, *p =* 0.59. **S8**: all naturally selected patches: 42 of 87, *p =* 0.67, dorsal naturally selected patches: 19 of 37, *p =* 0.5, ventral naturally selected patches: 23 of 50, *p =* 0.76, sexually selected patches: 7 of 18, *p =* 0.24. Figure 3a–c).

We further compared the distributions of the coefficients of variation between males and females and confirmed that variation of colour patches did not differ between the sexes (Wilcoxon signed-rank test: **B1**: naturally selected dorsal patches: V = 385, *p =* 0.62, *r* = 0.08, naturally selected ventral patches: V = 743, *p =* 0.31, *r* = 0.14, sexually selected patches: V = 120, *p =* 0.14, *r* = 0.35, **H3**: naturally selected dorsal patterns: V = 462, *p =* 0.09, *r* = 0.27, naturally selected ventral patches: V = 760, *p =* 0.24, *r* = 0.16, sexually selected patches: V = 114, *p =* 0.29, *r* = 0.29, **S8**: naturally selected dorsal patches: V = 319, *p =* 0.63, *r* = 0.08, naturally selected ventral patches: V = 555, *p =* 0.43, *r* = 0.11, sexually selected patches: V = 71, *p =* 0.55, *r* = 0.15).

To test whether brightness, hue, and saturation vary between males and females when specific functional roles of colour patches are considered, we further separated the naturally selected colours into: (a) aposematic, (b) mimetic, (c) cryptic, and (d) thermoregulatory colours (based on table S1). We also compared coefficients of variation for non-specific colour patches. These comparisons showed that there were no significant differences in the amount of variation in colour patches between males and females for any of the three colour parameters across the different functional classifications of colour patches and effect sizes were predominantly small (Table S5).

We also tested for colour-specific differences in the amount of variation by comparing coefficients of variation between males and females along the dorsoventral axis for black, brown, white, yellow, and red-orange colours. We specifically tested whether these colour patches were more variable in males than in females. We found that coefficients of variation were significantly higher in males only in one case concerning the hue for black on the dorsal surface. There were no significant differences between males and females in any other comparisons with predominantly small effect sizes (Table S6). Thus, males and females do not differ with respect to the amount of variation in spectral parameters of wing colour patches regardless of functional roles, wing surfaces, or colour identity.

#### Variation in relation to wing surfaces

We compared coefficients of variation between the dorsal and ventral surfaces within each sex for colour patches that were used in aposematism, mimicry, thermoregulation, and for colour patches that did not have specific known functions. There were no differences in variation between the dorsal and ventral surfaces in either sex for any functional class across the spectral parameters. We then separated the data by colour as done above and compared variability between the two wing surfaces within each colour. We did not find significant differences in coefficients of variation between dorsal and ventral surfaces for any colour and effect sizes were predominantly small (Table S7).

#### Variation in relation to functional roles

**Fig. 4:**
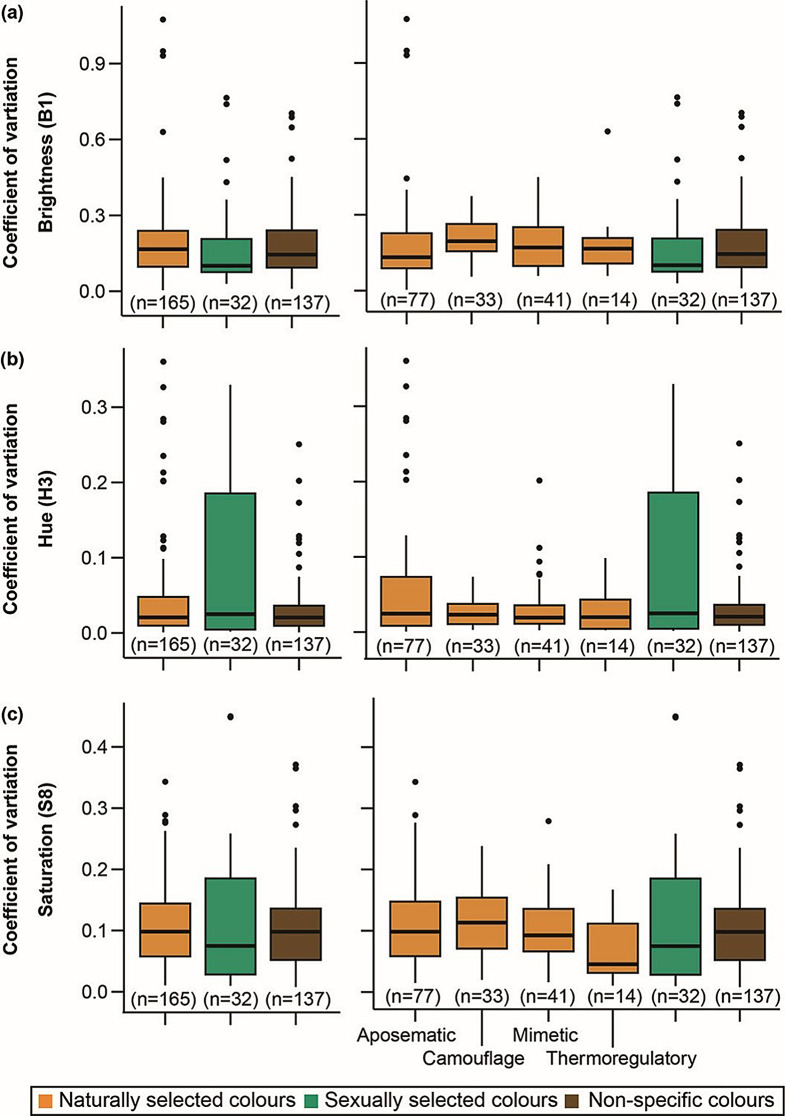
Boxplots of coefficients of variation for brightness **(a)**, hue **(b)**, and saturation **(c)** for colour patches combined according to ecological and sexual functions

To test whether the spectral parameters of colour patches shaped by sexual selection have greater variability than colour patches shaped by natural selection, we compared coefficients of variation of colour patches with different functions independent of sex and surface. We also kept non-specific colours in this comparison to study if variability is randomly distributed. We found that variation did not differ between the three groups for brightness, hue, or saturation (Kruskal-Wallis tests: **B1**: X^2^=3.525, df = 2, *p =* 0.17, effect size (η^2^) = 0.005, **H3**: X^2^=1.02, df = 2, *p =* 0.6, effect size (η^2^) = -0.0030, **S8**: X^2^=1.06, df = 2, *p =* 0.59, effect size (η^2^) =-0.0028). We also compared the coefficients of variation across finer classification into ecological functions of aposematism, mimicry, thermoregulation, camouflage, and sexual signalling to identify differences in the amount of variation, if any. We expected some functional categories such as camouflage and sexual signals to be more variable. However, there were no significant differences in the amount of variation between functional roles (Kruskal-Wallis tests: **B1**: X^2^=9.23, df = 5, *p =* 0.1, effect size (η^2^) = 0.013 **H3**: X^2^=2.69, df = 5, *p =* 0.75, effect size (η^2^) =-0.007, S**8**: X^2^=7.26, df = 5, *p =* 0.2, effect size (η^2^) = 0.007) (Fig. 4a–c).

## Discussion

Understanding the nature of variation provides insights into long-term dynamical equilibrium under which animal colour signals evolve [79]. By measuring reflectance spectra of wing colours across twenty butterfly species displaying diverse functions and amount of variation, we broaden the study of butterfly wing colour variation. We found that in the sample of species we measured, flashy/more conspicuous colour pattern elements such as iridescent colours, or reds and yellow were brighter and more saturated in males, and dull black/brown colouration was brighter in females and darker in males. This is suggestive of sexual selection on males for brighter pattern elements and more saturated colour patches on darker wing backgrounds, which may produce more conspicuous sexual signals when the sexes differ, depending on visual sensitivities of their conspecifics. However, it is striking that sexual differences did not exist in the majority of comparisons (usually more than 70% of the comparisons) (Fig. 2a). This general pattern indicated that in most species and colour patches measured, both the sexes showed comparable trait values for both naturally and sexually selected colour traits. Directional selection acting on traits is known to cause a shift in trait optima especially under the regime of sexual selection where mate choice might cause shifts towards brighter or more saturated colour signals [40, 72, 80]. Furthermore, in cases where there may not be a clear trend, differences between males and females across these colours may be explained by examining specific functions of the patches in specific contexts. However, how within-colour variability is affected while populations are shifting under these selective processes is not clear.

Contrary to our expectations, the comparison of variation between the sexes using coefficients of variation revealed that males and females do not, in most cases, differ in the amount of variation contained in the colour patches, regardless of colour identity, function, or wing surface (Supplementary Tables S5, S6). Previous works have suggested that: (a) females, especially of Lepidoptera, are more variable in colour and wing pattern polymorphisms such as those seen in Batesian mimicry, than males [52, 81–85], and (b) the degree of variation in female colour phenotypes as well as life history traits (e.g., ovarian dynamics associated with migration/dispersal, and thermal melanism) responds much more readily in a context-dependent manner to differential selection pressures, than that seen in males [42, 53–55, 86, 87]. Our exploratory study reveals that even in the presence of mean differences in spectral parameters of different colour patches of males and females, variability is not different between functionally different patches between the sexes. This is perhaps due to the strength of stabilising or directional selection in reducing spectral trait variation for the specific colour patches and species that we studied (Fig. 1, Table S1). Although our study uses a limited number of individuals, species, and wing patches which can restrict the conclusions drawn herein, it is informative and provides a basis to address fundamental evolutionary questions on trait variation in butterflies. These patterns need to be studied further with a larger dataset of individuals and species.

We also compared colours across the dorsoventral axis within each sex to characterise differences in trait values and trait variation. Dorsoventral comparisons showed a relatively higher number of differences than male-female comparisons. Nonetheless, surface-specific differences were also found only in a subset of comparisons. Where differences existed, we found that dorsal surfaces were brighter only for white and yellow colour patches. However, colour patches on the dorsal surface were more saturated than those on the ventral surface (Fig. 2b). Butterfly wing surfaces are also under differential selective pressures, with the dorsal surface more commonly used during courtship and the ventral surface for protective colouration [56, 88] though this is not always the case [89]. Therefore, potentially, differential selective pressures shape colour patches differently along the dorsoventral axis. However, we did not find evidence for higher variability of one surface compared to the other (Table S7). Finally, we also showed that despite colour patches having different functions, variation did not differ between aposematic, mimetic, thermoregulatory, cryptic, or sexually selected colour patches (Fig. 4). This contrasts with studies across bird species, where condition-dependent conspicuous [29] and sexual plumage were found to be more variable than non-sexual plumage [30]. While our classification of patch functions is grounded in existing literature, the lack of observed differences may also be attributed to insufficient comprehensive empirical studies on the precise roles of various colour patches in many butterfly species, especially when compared to the more extensive research conducted on birds.

Although our analysis with this subset of species did not support differences in variability across the comparisons we made, our results suggest that while trait differences exist, trait variation might be constrained to the same range in all colours across function; potentially highlighting that similar strength of selection shapes variation even if the mode of selection differs. Studies with more species and individuals can bolster these patterns highlighted here. Further, on estimating the effect sizes for the comparisons of coefficients of variation pooled across species (Tables S5–7), we found that most effect sizes were predominantly small, with a few that were moderate. With small effects, to achieve high enough power in any analysis, we further reiterate the need for larger samples sizes of individuals measured along with measuring more species [90]. Although, a recent study in moths with 20–30 individuals across 82 species digitally sampled [91] also reported similar small effect sizes and lack of significant differences for variation of colour metrics in relation to two antipredator strategies, along with other hypotheses tested. Therefore, our study still proves valuable by considering actual spectral variation across multiple functional roles of wing colour patches across 20 butterfly species. In cases where there were species-specific differences, specific functional tests and behavioural experiments might be required to determine significant effects of variability in colour patches. Further, future work needs to focus on testing whether this lack of differences in spectral variation is also functionally correlated within species when colours are viewed by conspecifics and heterospecifics through behavioural experiments or visual modelling. These subtle differences in colour can result in compartmentalizing colouration for functions such as predator avoidance or mate choice [92] which can, in turn, affect variation differently across species, sex, and wing surface. Additionally, chromatic and achromatic differences based on colour variation can also be channelled separately to accommodate both naturally and sexually selected functions of patches [93, 94]. Taken together, our work provides a basis for future studies to explore not only patterns in trait variation but also to empirically test functions of different signalling elements present on butterfly wings.

Adaptation of a population to novel or changing environments is often affected by the amount of variation contained among individuals of that population. Similar to standing genetic variation, standing phenotypic variation in populations should also facilitate faster evolution in the face of changing selective pressures [95]. However, we showed, in these species, that some wing colour patterns differed between the sexes indicating a differential response to sex-specific selection. But the lack of differences in the amount of variation between the sexes may act to reduce the rate at which sexes respond to changing climates. Interestingly, females do still tend to show more diversity in wing patterns than males [52, 81, 87] contributing to the idea that evolutionary trajectories of adaption between the sexes differ. Whether this is due to strong sexual selection on male colouration by females which constrains male adaptation even in changing environments and allows for what may appear to be faster rates of trait evolution in females is to be explored. However, changes in optima of female colouration may result in corresponding changes in optima of male colouration and vice-versa due to genetic correlations between the sexes. Thus, future studies can explore whether differences in mean values of colour patterns in response to differential selection pressures and the lack of differences in the amount of variation between the sexes constrain sex-specific adaptive responses to rapidly changing climate and human-dominated landscapes.

## Conclusions

This study describes spectral differences and variability of butterfly wing colouration used in different functional contexts in a sex and surface-specific manner. Our findings show that male butterflies often exhibit brighter, and more saturated colours compared to females, while dorsal surfaces display higher colour saturation than ventral surfaces. Interestingly, brightness variations between dorsal and ventral surfaces differed depending on the specific colour examined (Figs. 2, S2). However, despite differences in mean values, we reveal that the amount of variation across sex, surface, and functional roles did not differ (Figs. 3 and 4). This highlights an interesting pattern of colour variation across butterflies which can be further explored using experimental methods to elucidate the strength and mode of selection acting on these crucial visual signals. These patterns also provide insights contrary to studies in other taxa [29, 30, 91] though these studies use larger datasets. Further, in line with Nokelainen et al. (2024) [91] who found differences in wing pattern variation but no significant differences in colour metrics (brightness, hue, and saturation) in relation to antipredator strategies, our results also show similar effect sizes and lack of differences in spectral parameter variation across different functional roles. Larger samples sizes might uncover smaller effects of sex, or surface on colour variation and would improve the power of the tests conducted herein. This study opens multiple avenues of research that can investigate the eco-evolutionary mechanisms that shape or maintain butterfly wing variation.

## Electronic supplementary material

Below is the link to the electronic supplementary material.

Supplementary Material 1

Supplementary Material 2

## Data Availability

All data associated with this paper are available as Supplementary Tables in the supplementary files of the paper.

## References

[1] Mallarino R, Linden TA, Linnen CR, Hoekstra HE. The role of isoforms in the evolution of cryptic coloration in *Peromyscus* mice. Mol Ecol. 2017;26:245–58. 27105018 10.1111/mec.13663

[2] Dunn PO, Armenta JK, Whittingham LA. Natural and sexual selection act on different axes of variation in avian plumage color. Sci Adv. 2015;1. 10.1126/sciadv.1400155 PMC4643820 26601146

[3] Maan ME, Seehausen O. Ecology, sexual selection and speciation. Ecol Lett. 2011;14:591–602. 21375683 10.1111/j.1461-0248.2011.01606.x

[4] Linnen CR, Hoekstra HE. Measuring natural selection on genotypes and phenotypes in the wild. Cold Spring Harbor Symposia on quantitative Biology. NIH Public Access; 2009. pp. 155–68. 10.1101/sqb.2009.74.045 PMC3918505 20413707

[5] Gibbs HL, Grant PR. Oscillating selection on Darwin’s finches. Nature. 1987;327:511–3.

[6] Valvo JJ, Rodd FH, Hughes KA. Consistent female preference for rare and unfamiliar male color patterns in wild guppy populations. Behav Ecol. 2019;30:1672–81.

[7] Tanner JC, Swanger E, Zuk M. Sexual signal loss in field crickets maintained despite strong sexual selection favoring singing males. Evol (N Y). 2019;73:1482–9. 10.1111/evo.13761 31243769

[8] Heinen-Kay JL, Zuk M. When does sexual Signal Exploitation lead to Signal loss? Front Ecol Evol. 2019;7.

[9] Kingsolver JG, Pfennig DW. Patterns and power of phenotypic selection in Nature. Bioscience. 2007;57:561–72.

[10] Brodie ED III. Phenotypic selection on quantitative traits. The Princeton Guide to Evolution. Princeton University Press; 2014. pp. 221–9.

[11] Pomiankowski A, Iwasa Y. Runaway ornament diversity caused by fisherian sexual selection. Proc Natl Acad Sci U S A. 1998;95:5106–11. 9560236 10.1073/pnas.95.9.5106 PMC20221

[12] Henshaw JM, Jones AG. Fisher’s lost model of runaway sexual selection. Evol (N Y). 2020;74:487–94. 10.1111/evo.13910 31886520

[13] Hoekstra HE, Hoekstra JM, Berrigan D, Vignieri SN, Hoang A, Hill CE, et al. Strength and tempo of directional selection in the wild. Proc Natl Acad Sci U S A. 2001;98:9157–60. 11470913 10.1073/pnas.161281098 PMC55389

[14] Hosken DJ, House CM. Sexual selection. Curr Biol. 2011;21:R62–5. 21256434 10.1016/j.cub.2010.11.053

[15] Cameron E, Day T, Rowe L. Sexual conflict and indirect benefits. J Evol Biol. 2003;16:1055–60. 14635921 10.1046/j.1420-9101.2003.00584.x

[16] Rowe L, Houle D. The lek paradox and the capture of genetic variance by condition dependent traits. Proc R Soc B Biol Sci. 1996;263:1415–21.

[17] Cotton S, Fowler K, Pomiankowski A. Do sexual ornaments demonstrate heightened condition-dependent expression as predicted by the handicap hypothesis? Proc Royal Soc B: Biol Sci. 2004;271:771–83. 10.1098/rspb.2004.2688 PMC1691662 15255094

[18] Bonduriansky R. The evolution of condition-dependent sexual dimorphism. Am Nat. 2007;169:9–19. 17206580 10.1086/510214

[19] David P, Bjorksten T, Fowler K, Pomiankowski A. Condition-dependent signalling of genetic variation in stalk-eyed flies. Nature. 2000;406:186–8. 10910358 10.1038/35018079

[20] Cotton S, Fowler K, Pomiankowski A. Condition dependence of sexual ornament size and variation in the stalk-eyed fly *Cyrtodiopsis Dalmanni* (diptera: diopsidae). Evol (N Y). 2004;58:1038–46. 10.1111/j.0014-3820.2004.tb00437.x 15212384

[21] Harper GR, Pfennig DW, Harper GR Jr., Pfennig DW, Harper GR, Pfennig DW. Mimicry on the edge: why do mimics vary in resemblance to their model in different parts of their geographical range? Proc Biol Sci. 2007;274:1955–61. 17567563 10.1098/rspb.2007.0558 PMC2275182

[22] Kikuchi DW, Pfennig DW. High-model abundance may permit the gradual evolution of batesian mimicry: an experimental test. Proc R Soc B. 2010;277:1041–8. 19955153 10.1098/rspb.2009.2000 PMC2842773

[23] Akcali CK, Pfennig DW. Rapid evolution of mimicry following local model extinction. Biol Lett. 2014;10. 10.1098/rsbl.2014.0304 PMC4090552 24919704

[24] Caley MJ, Schluter D. Predators favour mimicry in a tropical reef fish. Proc R Soc B Biol Sci. 2003;270:667–72. 10.1098/rspb.2002.2263 PMC1691296 12713739

[25] Pfennig DW, Harcombe WR, Pfennig KS. Frequency-dependent batesian mimicry. Nature. 2001;410:323. 11268195 10.1038/35066628

[26] Hugall AF, Stuart-Fox D. Accelerated speciation in colour-polymorphic birds. Nature. 2012;485:631–4. 22660325 10.1038/nature11050

[27] Peters A, Delhey K, Andersson S, Van Noordwijk H, Förschler MI. Condition-dependence of multiple carotenoid‐based plumage traits: an experimental study. Funct Ecol. 2008;22:831–9.

[28] Johnsen A, Delhey K, Andersson S, Kempenaers B. Plumage colour in nestling blue tits: sexual dichromatism, condition dependence and genetic effects. Proc R Soc B Biol Sci. 2003;270:1263–70. 10.1098/rspb.2003.2375 PMC1691364 12816639

[29] Delhey K, Szecsenyi B, Nakagawa S, Peters A. Conspicuous plumage colours are highly variable. Proc R Soc B Biol Sci. 2017;284. 10.1098/rspb.2016.2593 PMC5310046 28100823

[30] Delhey K, Peters A. Quantifying variability of Avian colours: are signalling traits more variable? PLoS ONE. 2008;3:e1689. 18301766 10.1371/journal.pone.0001689 PMC2253496

[31] Cogliati KM, Corkum LD, Doucet SM. Bluegill Coloration as a sexual ornament: evidence from Ontogeny, sexual Dichromatism, and Condition Dependence. Ethology. 2010;116:416–28.

[32] Taylor LA, Clark DL, McGraw KJ. Condition dependence of male display coloration in a jumping spider (*Habronattus pyrrithrix*). Behav Ecol Sociobiol. 2011;65:1133–46.

[33] Chouteau M, Arias M, Joron M. Warning signals are under positive frequency-dependent selection in nature. Proc Natl Acad Sci. 2016;113:2164–9. 26858416 10.1073/pnas.1519216113 PMC4776528

[34] Suzuki TK. Camouflage variations on a theme of the Nymphalid Ground Plan. Diversity and evolution of Butterfly Wing patterns. Singapore: Springer Singapore; 2017. pp. 39–58.

[35] Thurman TJ, Seymoure BM. A bird’s eye view of two mimetic tropical butterflies: coloration matches predator’s sensitivity. J Zool. 2016;298:159–68.

[36] Langham GMGM. Specialized avian predators repeatedly attack novel color morphs of *Heliconius* butterflies. Evol (N Y). 2004;58:2783–7. 10.1111/j.0014-3820.2004.tb01629.x 15696755

[37] Uesugi K. The adaptive significance of batesian mimicry in the swallowtail butterfly *Papilio polytes* (Insecta, Papilionidae): associative learning in a predator. Ethology. 1996;102:762–75.

[38] Fordyce JA, Nice CC, Forister ML, Shapiro AM. The significance of wing pattern diversity in the Lycaenidae: mate discrimination by two recently diverged species. J Evol Biol. 2002;15:871–9.

[39] Rutowski RL. Male-specific iridescent coloration in the pipevine swallowtail (*Battus philenor*) is used in mate choice by females but not sexual discrimination by males. J Insect Behav. 2013;26:200–11.

[40] Kemp DJ. Female butterflies prefer males bearing bright iridescent ornamentation. Proc R Soc B. 2007;274:1043–7. 17284412 10.1098/rspb.2006.0043 PMC2124467

[41] Rossi M, Hausmann AE, Alcami P, Moest M, Roussou R, Van Belleghem SM, et al. Adaptive introgression of a visual preference gene. Sci (80-). 2024;383:1368–73. 10.1126/science.adj9201 PMC7616200 38513020

[42] Gautam S, Kunte K. Adaptive plasticity in wing melanisation of a montane butterfly across a himalayan elevational gradient. Ecol Entomol. 2020;45:1272–83.

[43] Ellers J, Boggs CL. Functional ecological implications of intraspecific differences in wing melanization in *Colias* butterflies. Biol J Linn Soc. 2004;82:79–87.

[44] Shawkey MD, Morehouse NI, Vukusic P. A protean palette: Colour materials and mixing in birds and butterflies. J Royal Soc Interface. 2009;6 SUPPL. 2. 10.1098/rsif.2008.0459.focus PMC2706479 19141430

[45] White TE. Structural colours reflect individual quality: a meta-analysis. Biol Lett. 2020;16. 10.1098/rsbl.2020.0001 PMC7211460 32289245

[46] Kemp D. Resource-mediated condition dependence in sexually dichromatic butterfly wing coloration. Evol (N Y). 2008;62:2346–58. 10.1111/j.1558-5646.2008.00461.x 18637962

[47] Kemp DJ, Rutowski RL. Condition dependence, quantitative genetics, and the potential signal content of iridescent ultraviolet butterfly coloration. Evol (N Y). 2007;61:168–83. 10.1111/j.1558-5646.2007.00014.x 17300436

[48] Morehouse NI, Vukusic P, Rutowski R. Pterin pigment granules are responsible for both broadband light scattering and wavelength selective absorption in the wing scales of pierid butterflies. Proc R Soc B Biol Sci. 2007;274:359–66. 10.1098/rspb.2006.3730 PMC1702378 17164199

[49] Giraldo MA, Stavenga DG. Sexual dichroism and pigment localization in the wing scales of *Pieris rapae* butterflies. Proc R Soc B. 2007;274:97–102. 17018427 10.1098/rspb.2006.3708 PMC1679869

[50] Pegram KV, Nahm AC, Rutowski RL. Warning Color changes in response to Food Deprivation in the Pipevine Swallowtail Butterfly, *Battus philenor*. J Insect Sci. 2013;13:1–16. 24735188 10.1673/031.013.11001 PMC4011348

[51] Talloen W, Dyck H, Van, Lens L. The cost of melanization: butterfly wing coloration under environmental stress. Evol (N Y). 2004;58:360–6. 10.1111/j.0014-3820.2004.tb01651.x 15068352

[52] Fisher RA, Ford EB. The variability of species in the Lepidoptera, with reference to abundance and sex. Trans Entomol Soc Lond. 1929;15:19:367–84.

[53] Basu DN, Bhaumik V, Kunte K. The tempo and mode of character evolution in the assembly of mimetic communities. Proc Natl Acad Sci USA. 2023;120:e2203724120. 36577073 10.1073/pnas.2203724120 PMC9910590

[54] Bhaumik V, Kunte K. Female butterflies modulate investment in reproduction and flight in response to monsoon-driven migrations. Oikos. 2018;127:285–96.

[55] Bhaumik V, Kunte K. Dispersal and migration have contrasting effects on butterfly flight morphology and reproduction. Biol Lett. 2020;16:20200393. 32810429 10.1098/rsbl.2020.0393 PMC7480162

[56] Oliver JC, Robertson KA, Monteiro A. Accommodating natural and sexual selection in butterfly wing pattern evolution. Proc R Soc B. 2009;276:2369–75. 19364741 10.1098/rspb.2009.0182 PMC2690465

[57] Silberglied RE. Visual communication and sexual selection among butterflies. In: Vane-Wright RI, Ackery PR, editors. The Biology of butterflies. London: Academic; 1984. pp. 207–23.

[58] Codella SG Jr, Lederhouse RC. Intersexual comparison of mimetic protection in the black swallowtail butterfly, *Papilio polyxenes*: experiments with captive blue jay predators. Evol (N Y). 1989;43:410–20. 10.1111/j.1558-5646.1989.tb04236.x 28568560

[59] Su S, Lim M, Kunte K. Prey from the eyes of predators: Color discriminability of aposematic and mimetic butterflies from an avian visual perspective. Evol (N Y). 2015;69:2985–94. 10.1111/evo.12800 26477885

[60] Dalrymple RL, Hui FKC, Flores-Moreno H, Kemp DJ, Moles AT. Roses are red, violets are blue - so how much replication should you do? An assessment of variation in the colour of flowers and birds. Biol J Linn Soc. 2015;114:69–81.

[61] Joshi J, Prakash A, Kunte K. Evolutionary assembly of communities in butterfly mimicry rings. Am Nat. 2017;189:E58–76. 28350498 10.1086/690907

[62] Brakefield PM. Tropical dry and wet season polyphenism in the butterfly *Melanitis leda* (Satyrinae): phenotypic plasticity and climatic correlates. Biol J Linn Soc. 1987;31:175–91.

[63] Brakefield PM. Crypsis. Encyclopedia of insects. Elsevier Inc.; 2009. pp. 236–9.

[64] Dharmaraaj B, Venkatesan R, Kunte K. Spectral variation and pigmentary basis of ornamental and mimetic wing colour patches of swallowtail butterflies. Evol J Linn Soc. 2024. 10.1093/evolinnean/kzae018.

[65] Wee JLQ, Monteiro A. Yellow and the novel aposematic signal, red, protect *Delias* butterflies from predators. PLoS ONE. 2017;12:e0168243. 28060944 10.1371/journal.pone.0168243 PMC5218396

[66] Silberglied RE. Communication in the ultraviolet. Annu Rev Ecol Syst. 1979;10:373–98.

[67] Silberglied RE, Taylor OR. Ultraviolet Reflection and its behavioral role in the courtship of the sulfur butterflies *Colias eurytheme* and *C. philodice* (Lepidoptera, Pieridae). Behav Ecol Sociobiol. 1978;3:203–43.

[68] Papke RS, Kemp DJ, Rutowski RL. Multimodal signalling: structural ultraviolet reflectance predicts male mating success better than pheromones in the butterfly *Colias eurytheme* L. (Pieridae). Anim Behav. 2007;73:47–54.

[69] Kemp DJ. Female mating biases for bright ultraviolet iridescence in the butterfly *Eurema hecabe *(Pieridae). Behav Ecol. 2008;19:1–8.

[70] Ficarrotta V, Martin A, Counterman BA, Pyron RA. Early origin and diverse phenotypic implementation of iridescent UV patterns for sexual signaling in pierid butterflies. Evol (N Y). 2023;77:2619–30. 10.1093/evolut/qpad174 37797261

[71] Sasaki N, Konagaya T, Watanabe M, Rutowski RL. Estimating the mating success of male butterflies in the field. In: Sekimura T, Nijhout HF, editors. Diversity and Evolution of Butterfly Wing Patterns. 1st edition. Singapore: Springer Singapore; 2017. pp. 255–68.

[72] Maan ME, Cummings ME. Sexual dimorphism and directional sexual selection on aposematic signals in a poison frog. Proc Natl Acad Sci U S A. 2009;106:19072–7. 19858491 10.1073/pnas.0903327106 PMC2776464

[73] Ellers J, Boggs CL. The evolution of wing color: male mate choice opposes adaptive wing color divergence in *Colias* butterflies. Evol (N Y). 2003;57:1100–6. 10.1111/j.0014-3820.2003.tb00319.x 12836826

[74] Finkbeiner SD, Briscoe AD, Reed RD. Warning signals are seductive: relative contributions of color and pattern to predator avoidance and mate attraction in *Heliconius* butterflies. Evol (N Y). 2014;68:3410–20. 10.1111/evo.12524 25200939

[75] Maia R, Gruson H, Endler JA, White TE. pavo 2: New tools for the spectral and spatial analysis of colour in R. Methods Ecol Evol. 2019;10:1097–107.

[76] R Development Core Team. R: a language and environment for statistical computing. 2019.

[77] Lenth R, Singmann H, Love J, Buerkner P, Herve M. emmeans: estimated marginal means, aka least-squares means. R package version 1.4.5. 2019.

[78] Kassambara A. Pipe-Friendly Framework for Basic Statistical Tests [R package rstatix version 0.7.2]. 2023.

[79] Cuthill IC, Allen WL, Arbuckle K, Caspers B, Chaplin G, Hauber ME et al. The biology of color. Science. 2017;357. 10.1126/science.aan0221 28774901

[80] Pauers MJ, McKinnon JS, Ehlinger TJ. Directional sexual selection on chroma and within-pattern colour contrast in *Labeotropheus fuelleborni*. Proc R Soc B Biol Sci. 2004;271 SUPPL. 6. 10.1098/rsbl.2004.0215 PMC1810103 15801599

[81] Pearse FK, Murray ND. Sex and variability in the common brown butterfly *Heteronympha merope merope* (Lepidoptera: Satyrinae). Evol (N Y). 1982;36:1251–64. 10.1111/j.1558-5646.1982.tb05494.x 28563570

[82] Hazel WN. Sex-limited variability and mimicry in the swallowtail butterfly *Papilio polyxenes* Fabr. Heredity (Edinb). 1990;65:109–14.

[83] Kunte K. Mimetic butterflies support Wallace’s model of sexual dimorphism. Proc R Soc B. 2008;275:1617–24. 18426753 10.1098/rspb.2008.0171 PMC2602815

[84] Kunte K. Female-limited mimetic polymorphism: a review of theories and a critique of sexual selection as balancing selection. Anim Behav. 2009;78:1029–36.

[85] Kunte K. The diversity and evolution of batesian mimicry in *Papilio* swallowtail butterflies. Evol (N Y). 2009;63:2707–16. 10.1111/j.1558-5646.2009.00752.x 19552738

[86] Tunström K, Woronik A, Hanly JJ, Rastas P, Chichvarkhin A, Warren AD et al. Evidence for a single, ancient origin of a genus-wide alternative life history strategy. Sci Adv. 2023;9. 10.1126/sciadv.abq3713 PMC10032607 36947619

[87] Tuomaala M, Kaitala A, Rutowski RL. Females show greater changes in wing colour with latitude than males in the green-veined white butterfly, *Pieris napi* (Lepidoptera: Pieridae). Biol J Linn Soc. 2012;107:899–909.

[88] Rutowski RL, Nahm AC, Macedonia JM. Iridescent hindwing patches in the Pipevine Swallowtail: differences in dorsal and ventral surfaces relate to signal function and context. Funct Ecol. 2010;24:767–75.

[89] Huq M, Bhardwaj S, Monteiro A. Male *Bicyclus anynana* butterflies choose females on the basis of their ventral UV-reflective eyespot centers. J Insect Sci. 2019;19. 10.1093/jisesa/iez014 PMC6390274 30794728

[90] Serdar CC, Cihan M, Yücel D, Serdar MA. Sample size, power and effect size revisited: simplified and practical approachin pre-clinical, clinical and laboratory studies. Biochem Med. 2021;31:1–27. 10.11613/BM.2021.010502 PMC7745163 33380887

[91] Nokelainen O, Silvasti SA, Strauss SY, Wahlberg N, Mappes J. Predator selection on phenotypic variability of cryptic and aposematic moths. Nat Commun. 2024;15:1–12. 38395999 10.1038/s41467-024-45329-5 PMC10891176

[92] Dell’Aglio DD, Troscianko J, McMillan WO, Stevens M, Jiggins CD. The appearance of mimetic *Heliconius* butterflies to predators and conspecifics. Evolution (N Y). 2018;72:2156–66. 10.1111/evo.13583 PMC6221148 30129174

[93] Dalbosco D, Aglio D’, Troscianko J, Stevens M, Mcmillan O, Jiggins CD. The conspicuousness of the toxic *Heliconius* butterflies across time and habitat. bioRxiv. 2020;662155.

[94] Dang A, Bernard GD, Yuan F, Macias-Muñoz A, Hill RI, Lawrence JP et al. Mimetic butterfly wings through mimetic butterfly eyes: Evidence that brightness vision helps *Adelpha fessonia* identify potential mates. bioRxiv. 2023;:2023.10.05.561098.

[95] Barrett RDH, Schluter D. Adaptation from standing genetic variation. Trends Ecol Evol. 2008;23:38–44. 18006185 10.1016/j.tree.2007.09.008

