# Supplementary material for: Natural and sexual selection and functional roles influence colouration but not the amount of variation in butterfly wing colour patterns
Source: BMC Ecol Evol. 2025 Jan 17;25:11. doi: 10.1186/s12862-024-02346-8 (PMC11740640; doi:10.1186/s12862-024-02346-8)
Supplement: Supplementary file 2 — Supplementary Material 2 [file 12862_2024_2346_MOESM2_ESM.docx]

**Supplementary Tables and Figures**

**Table S1:** List of species and number of individuals for which reflectance spectra were measured. Asterisk (*) indicates references that directly highlight functions of colour patches. References without asterisks are functions inferred from larger, global patterns from species with similar phenotypes or studies from related species. Corresponding colours measured and butterfly images can be found in Fig S1.

| Sl.No. | Species | Family | Males | Females | Primary Ecological functions | Colours considered | References |
| --- | --- | --- | --- | --- | --- | --- | --- |
| 1 | *Pachliopta aristolochiae* | Papilionidae | 5 | 3 | Aposematic | All wing colours | 61*,53*,59* |
| 2 | *Byasa latreillei* | Papilionidae | 4 | 5 | Aposematic | All wing colours | 61* |
| 3 | *Cethosia biblis* | Nymphalidae | 5 | 6 | 1) Aposematic  2) Putative sexual signal in males | 1) All wing colours  2) Dorsal red colours | 61* |
| 4 | *Papilio clytia* | Papilionidae | 6 | 6 | Mimetic | All wing colours | 61*,53*,59* |
| 5 | *Colias fieldii* | Pieridae | 7 | 7 | Sexual signal: UV-reflective colour patch | Dorsal yellow/orange colours | 66–70 |
| 6 | *Colotis danae* | Pieridae | 5 | 5 | Sexual signal:UV-reflective colour patch | Dorsal forewing red | 66–70 |
| 7 | *Delias descombesi* | Pieridae | 10 | 10 | Aposematic | All wing colours | 61*,65 |
| 8 | *Hebomoia glaucippe* | Pieridae | 6 | 4 | 1) Sexual signal:Dorsal UV-reflective colour patch  2) Cryptic ventral | 1) Dorsal forewing orange  2) All ventral colours | 66–70 |
| 9 | *Hypolimnas bolina* | Nymphalidae | 7 | 5 | 1) Mimetic females  2) Sexual signal: Iridescent white patch in males | 1) All wing colours in females are mimetic  2) Iridescent dorsal white in males | 61*, 53*,59*,40* |
| 10 | *Ixias pyrene* | Pieridae | 10 | 8 | Sexual signal: UV-reflective colour patch | Dorsal forewing orange | 66–70 |
| 11 | *Junonia hierta* | Nymphalidae | 10 | 7 | Sexual signal: Iridescent blue patch | Dorsal hindwing blue | 57, 39 |
| 12 | *Melanitis leda* (dry season form) | Nymphalidae | 6 | 4 | Cryptic ventral | Ventral brown colour | 62*,63* |
| 13 | *Papilio helenus* | Papilionidae | 8 | 4 | Sexual signal: Fluorescent white patch | Dorsal hindwing white | 61, 53, 64* |
| 14 | *Tajuria cippus* | Lycaenidae | 8 | 8 | Sexual signal: Iridescent blue patch | Dorsal fore-and hindwing blue colours | 57, 39 |
| 15 | *Papilio demoleus* | Papilionidae | 10 | 10 | Sexual signal: Fluorescent white patch | Dorsal fore and hindwing yellow colours | 61, 53, 64* |
| 16 | *Doleschallia bisaltide* | Nymphalidae | 5 | 3 | Cryptic ventral | Ventral brown colour | 63 |
| 17 | *Elymnias caudata* | Nymphalidae | 10 | 10 | 1) Mimetic female dorsal  2) Putative sexual signal in males  3) Cryptic ventral | 1) Mimetic females: dorsal fore and hindwing  2) dorsal brown colouration  3) all ventral colours in males and females | 61*,63,53*,59* |
| 18 | *Pareronia hippia* | Pieridae | 10 | 10 | 1) Mimetic females  2) Nonmimetic, sexually selected males | 1) Mimetic females: all wing colours  2) Non-mimetic males: dorsal fore and hindwing blue-white colours | 61*,53*,59* |
| 19 | *Pieris canidia* | Pieridae | 10 | 10 | Thermoregulatory | All wing colours | 42* |
| 20 | *Vanessa indica* | Nymphalidae | 6 | 6 | Cryptic ventral | All ventral brown colours | 63 |

**Table S2: Reflectance spectra values for species and specimens used in this work.** See Excel sheet, ‘TableS2_ReflectanceSpectra.xlsx’.

**Table S3:** GLM incorporating Sex and Surface for spectral colour parameters for each species, separated by colour. Spectral colour parameters: Brightness (B1), Hue (H3, H1), and Saturation (S8, S1R, S1Y, S1B). Significant values are in bold. These results correspond to Fig. 2, and Fig. S2a–f.

| **Colour** | **Colour parameter** | **Species** | **Coefficients** | **Estimate** | **Std. Error** | **t value** | **Pr(>\|t\|)** |
| --- | --- | --- | --- | --- | --- | --- | --- |
| Black | B1 | *Pachliopta aristolochiae* | (Intercept) | 324.59 | 78.35 | 4.14 | **0.00** |
|  |  |  | Surface-Ventral | 190.15 | 110.80 | 1.72 | 0.10 |
|  |  |  | Sex-Female | 239.38 | 127.94 | 1.87 | 0.07 |
|  |  |  | Surface-Ventral:Sex-female | -181.31 | 180.94 | -1.00 | 0.32 |
|  |  | *Byasa latreillei* | (Intercept) | 517.91 | 139.00 | 3.73 | **0.00** |
|  |  |  | Surface-Ventral | 493.35 | 196.58 | 2.51 | **0.02** |
|  |  |  | Sex-Female | 37.01 | 186.49 | 0.20 | 0.84 |
|  |  |  | Surface-Ventral:Sex-female | -90.06 | 263.74 | -0.34 | 0.73 |
|  |  | *Colias fieldii* | (Intercept) | 1679.64 | 109.98 | 15.27 | **<2e-16** |
|  |  |  | Surface-Ventral | 92.71 | 190.49 | 0.49 | 0.63 |
|  |  |  | Sex-Female | -295.70 | 155.53 | -1.90 | 0.06 |
|  |  |  | Surface-Ventral:Sex-female | -10.88 | 269.39 | -0.04 | 0.97 |
|  |  | *Delias descombesi* | (Intercept) | 1318.10 | 240.10 | 5.49 | **0.00** |
|  |  |  | Surface-Ventral | -169.20 | 208.00 | -0.81 | 0.42 |
|  |  |  | Sex-Female | -200.60 | 169.80 | -1.18 | 0.24 |
|  |  | *Papilio demoleus* | (Intercept) | 180.93 | 52.80 | 3.43 | **0.00** |
|  |  |  | Surface-Ventral | 345.39 | 74.67 | 4.63 | **0.00** |
|  |  |  | Sex-Female | 169.39 | 74.67 | 2.27 | **0.03** |
|  |  |  | Surface-Ventral:Sex-female | -35.59 | 105.60 | -0.34 | 0.74 |
|  |  | *Papilio helenus* | (Intercept) | 251.11 | 51.93 | 4.84 | **0.00** |
|  |  |  | Surface-Ventral | 228.19 | 73.44 | 3.11 | **0.00** |
|  |  |  | Sex-Female | 90.23 | 89.95 | 1.00 | 0.32 |
|  |  |  | Surface-Ventral:Sex-female | 136.48 | 127.20 | 1.07 | 0.29 |
|  |  | *Pareronia hippia* | (Intercept) | 1035.00 | 189.90 | 5.45 | **0.00** |
|  |  |  | Surface-Ventral | 2306.30 | 268.50 | 8.59 | **0.00** |
|  |  |  | Sex-Female | 151.90 | 268.50 | 0.57 | 0.57 |
|  |  | *Pieris canidia* | (Intercept) | 2378.83 | 107.04 | 22.23 | **<2e-16** |
|  |  |  | Surface-Ventral | -76.04 | 192.14 | -0.40 | 0.69 |
|  |  |  | Sex-Female | 153.50 | 151.37 | 1.01 | 0.32 |
|  |  |  | Surface-Ventral:Sex-female | -18.35 | 266.99 | -0.07 | 0.95 |
|  |  | *Vanessa indica* | (Intercept) | 354.58 | 61.39 | 5.78 | **0.00** |
|  |  |  | Surface-Ventral | 255.91 | 86.82 | 2.95 | **0.01** |
|  |  |  | Sex-Female | 181.09 | 86.82 | 2.09 | 0.05 |
|  |  |  | Surface-Ventral:Sex-female | -207.51 | 122.78 | -1.69 | 0.11 |
|  | H3 | *Pachliopta aristolochiae* | (Intercept) | 651.00 | 4.80 | 135.69 | **< 2e-16** |
|  |  |  | Surface-Ventral | -18.20 | 6.79 | -2.68 | **0.01** |
|  |  |  | Sex-Female | -26.67 | 7.84 | -3.40 | **0.00** |
|  |  |  | Surface-Ventral:Sex-female | 19.87 | 11.08 | 1.79 | 0.08 |
|  |  | *Byasa latreillei* | (Intercept) | 568.00 | 17.40 | 32.65 | **<2e-16** |
|  |  |  | Surface-Ventral | 7.25 | 24.60 | 0.30 | 0.77 |
|  |  |  | Sex-Female | 45.80 | 23.34 | 1.96 | 0.06 |
|  |  |  | Surface-Ventral:Sex-female | -31.05 | 33.01 | -0.94 | 0.35 |
|  |  | *Colias fieldii* | (Intercept) | 604.50 | 3.93 | 153.67 | **<2e-16** |
|  |  |  | Surface-Ventral | 10.79 | 6.81 | 1.58 | 0.12 |
|  |  |  | Sex-Female | 9.79 | 5.56 | 1.76 | 0.09 |
|  |  |  | Surface-Ventral:Sex-female | -0.93 | 9.64 | -0.10 | 0.92 |
|  |  | *Delias descombesi* | (Intercept) | 617.35 | 12.99 | 47.51 | **< 2e-16** |
|  |  |  | Surface-Ventral | 2.50 | 11.25 | 0.22 | 0.83 |
|  |  |  | Sex-Female | 31.15 | 9.19 | 3.39 | **0.00** |
|  |  | *Papilio demoleus* | (Intercept) | 645.15 | 7.48 | 86.30 | **<2e-16** |
|  |  |  | Surface-Ventral | -7.45 | 10.57 | -0.71 | 0.48 |
|  |  |  | Sex-Female | -24.70 | 10.57 | -2.34 | **0.02** |
|  |  |  | Surface-Ventral:Sex-female | 21.30 | 14.95 | 1.43 | 0.16 |
|  |  | *Papilio helenus* | (Intercept) | 608.13 | 12.51 | 48.60 | **<2e-16** |
|  |  |  | Surface-Ventral | 34.06 | 17.70 | 1.93 | 0.06 |
|  |  |  | Sex-Female | 33.25 | 21.67 | 1.53 | 0.13 |
|  |  |  | Surface-Ventral:Sex-female | -38.06 | 30.65 | -1.24 | 0.22 |
|  |  | *Pareronia hippia* | (Intercept) | 611.00 | 4.32 | 141.41 | **< 2e-16** |
|  |  |  | Surface-Ventral | -39.85 | 6.11 | -6.52 | **0.00** |
|  |  |  | Sex-Female | 10.65 | 6.11 | 1.74 | 0.09 |
|  |  | *Pieris canidia* | (Intercept) | 572.70 | 5.96 | 96.11 | **<2e-16** |
|  |  |  | Surface-Ventral | 16.74 | 10.70 | 1.57 | 0.12 |
|  |  |  | Sex-Female | 11.30 | 8.43 | 1.34 | 0.19 |
|  |  |  | Surface-Ventral:Sex-female | -10.64 | 14.86 | -0.72 | 0.48 |
|  |  | *Vanessa indica* | (Intercept) | 595.33 | 6.25 | 95.33 | **<2e-16** |
|  |  |  | Surface-Ventral | 8.50 | 8.83 | 0.96 | 0.35 |
|  |  |  | Sex-Female | 33.00 | 8.83 | 3.74 | **0.00** |
|  |  |  | Surface-Ventral:Sex-female | -16.00 | 12.49 | -1.28 | 0.21 |
|  | S8 | *Pachliopta aristolochiae* | (Intercept) | 1.87 | 0.10 | 19.34 | **<2e-16** |
|  |  |  | Surface-Ventral | -0.01 | 0.14 | -0.06 | 0.95 |
|  |  |  | Sex-Female | 0.05 | 0.16 | 0.31 | 0.76 |
|  |  |  | Surface-Ventral:Sex-female | -0.04 | 0.22 | -0.16 | 0.88 |
|  |  | *Byasa latreillei* | (Intercept) | 1.12 | 0.08 | 14.50 | **0.00** |
|  |  |  | Surface-Ventral | 0.18 | 0.11 | 1.64 | 0.11 |
|  |  |  | Sex-Female | 0.00 | 0.10 | 0.04 | 0.97 |
|  |  |  | Surface-Ventral:Sex-female | -0.13 | 0.15 | -0.85 | 0.40 |
|  |  | *Colias fieldii* | (Intercept) | 1.95 | 0.05 | 40.28 | **< 2e-16** |
|  |  |  | Surface-Ventral | 0.19 | 0.08 | 2.31 | **0.03** |
|  |  |  | Sex-Female | 0.22 | 0.07 | 3.22 | **0.00** |
|  |  |  | Surface-Ventral:Sex-female | -0.29 | 0.12 | -2.47 | **0.02** |
|  |  | *Delias descombesi* | (Intercept) | 1.48 | 0.11 | 13.90 | **< 2e-16** |
|  |  |  | Surface-Ventral | 0.05 | 0.09 | 0.49 | 0.63 |
|  |  |  | Sex-Female | -0.29 | 0.08 | -3.85 | **0.00** |
|  |  | *Papilio demoleus* | (Intercept) | 2.89 | 0.15 | 19.15 | **<2e-16** |
|  |  |  | Surface-Ventral | -0.51 | 0.21 | -2.38 | **0.02** |
|  |  |  | Sex-Female | -0.15 | 0.21 | -0.72 | 0.47 |
|  |  |  | Surface-Ventral:Sex-female | 0.29 | 0.30 | 0.97 | 0.33 |
|  |  | *Papilio helenus* | (Intercept) | 2.56 | 0.11 | 22.56 | **<2e-16** |
|  |  |  | Surface-Ventral | -0.46 | 0.16 | -2.86 | **0.01** |
|  |  |  | Sex-Female | -0.01 | 0.20 | -0.07 | 0.94 |
|  |  |  | Surface-Ventral:Sex-female | -0.32 | 0.28 | -1.16 | 0.25 |
|  |  | *Pareronia hippia* | (Intercept) | 2.03 | 0.05 | 37.11 | **< 2e-16** |
|  |  |  | Surface-Ventral | -0.35 | 0.08 | -4.53 | **0.00** |
|  |  |  | Sex-Female | 0.48 | 0.08 | 6.25 | **0.00** |
|  |  | *Pieris canidia* | (Intercept) | 1.94 | 0.06 | 33.90 | **<2e-16** |
|  |  |  | Surface-Ventral | 0.19 | 0.10 | 1.85 | 0.07 |
|  |  |  | Sex-Female | 0.07 | 0.08 | 0.87 | 0.39 |
|  |  |  | Surface-Ventral:Sex-female | -0.32 | 0.14 | -2.24 | **0.03** |
|  |  | *Vanessa indica* | (Intercept) | 2.12 | 0.12 | 17.12 | **0.00** |
|  |  |  | Surface-Ventral | 0.51 | 0.17 | 2.92 | **0.01** |
|  |  |  | Sex-Female | 0.19 | 0.17 | 1.06 | 0.30 |
|  |  |  | Surface-Ventral:Sex-female | -0.07 | 0.25 | -0.30 | 0.77 |
| Brown | B1 | *Papilio clytia* | (Intercept) | 581.57 | 99.09 | 5.869 | **0.00** |
|  |  |  | Surface-Ventral | 1034.11 | 140.13 | 7.38 | **0.00** |
|  |  |  | Sex-Female | 497.72 | 140.13 | 3.552 | **0.00** |
|  |  |  | Surface-Ventral:Sex-female | 52.26 | 198.18 | 0.264 | 0.79 |
|  |  | *Doleschallia bisaltide* | (Intercept) | 3972.9 | 376.8 | 10.543 | **0.00** |
|  |  |  | Surface-Ventral | -929.5 | 461.5 | -2.014 | 0.05 |
|  |  |  | Sex-Female | 1013.3 | 615.3 | 1.647 | 0.11 |
|  |  |  | Surface-Ventral:Sex-female | 240.9 | 753.6 | 0.32 | 0.75 |
|  |  | *Elymnias caudata* | (Intercept) | 567.1 | 319.3 | 1.776 | 0.08 |
|  |  |  | Surface-Ventral | 2279.1 | 412.2 | 5.529 | **0.00** |
|  |  |  | Sex-Female | 315 | 451.5 | 0.698 | 0.49 |
|  |  |  | Surface-Ventral:Sex-female | 1982.7 | 582.9 | 3.401 | **0.00** |
|  |  | *Hypolimnas bolina* | (Intercept) | 497.08 | 101.82 | 4.882 | **0.00** |
|  |  |  | Surface-Ventral | 1350.22 | 143.99 | 9.377 | **0.00** |
|  |  |  | Sex-Female | 504.5 | 157.73 | 3.198 | **0.00** |
|  |  |  | Surface-Ventral:Sex-female | -14.35 | 223.07 | -0.064 | 0.95 |
|  |  | *Melanitis leda* | (Intercept) | 2508.43 | 263.13 | 9.533 | **0.00** |
|  |  |  | Surface-Ventral | 2395.64 | 372.12 | 6.438 | **0.00** |
|  |  |  | Sex-Female | 68.49 | 455.76 | 0.15 | 0.88 |
|  |  |  | Surface-Ventral:Sex-female | 287.91 | 644.54 | 0.447 | 0.66 |
|  |  | *Vanessa indica* | (Intercept) | 1543.46 | 176.04 | 8.768 | **0.00** |
|  |  |  | Surface-Ventral | -64.23 | 304.9 | -0.211 | 0.83 |
|  |  |  | Sex-Female | 673.24 | 248.95 | 2.704 | **0.01** |
|  |  |  | Surface-Ventral:Sex-female | 52.79 | 431.2 | 0.122 | 0.90 |
|  | H3 | *Papilio clytia* | (Intercept) | 643.833 | 2.742 | 234.823 | **< 2e-16** |
|  |  |  | Surface-Ventral | -19.917 | 3.877 | -5.137 | **0.00** |
|  |  |  | Sex-Female | -18.25 | 3.877 | -4.707 | **0.00** |
|  |  |  | Surface-Ventral:Sex-female | 5.167 | 5.484 | 0.942 | 0.35 |
|  |  | *Doleschallia bisaltide* | (Intercept) | 565.8 | 2.317 | 244.246 | **< 2e-16** |
|  |  |  | Surface-Ventral | 36.35 | 2.837 | 12.812 | **< 2e-16** |
|  |  |  | Sex-Female | -12.8 | 3.783 | -3.384 | **0.00** |
|  |  |  | Surface-Ventral:Sex-female | 3.15 | 4.633 | 0.68 | 0.50 |
|  |  | *Elymnias caudata* | (Intercept) | 638.95 | 3.614 | 176.783 | **< 2e-16** |
|  |  |  | Surface-Ventral | -30.95 | 4.666 | -6.633 | **0.00** |
|  |  |  | Sex-Female | -6.2 | 5.111 | -1.213 | 0.23 |
|  |  |  | Surface-Ventral:Sex-female | -31.767 | 6.599 | -4.814 | **0.00** |
|  |  | *Hypolimnas bolina* | (Intercept) | 630.5 | 3.645 | 172.996 | **< 2e-16** |
|  |  |  | Surface-Ventral | -23.286 | 5.154 | -4.518 | **0.00** |
|  |  |  | Sex-Female | -0.3 | 5.646 | -0.053 | 0.96 |
|  |  |  | Surface-Ventral:Sex-female | -2.814 | 7.985 | -0.352 | 0.73 |
|  |  | *Melanitis leda* | (Intercept) | 598.9167 | 4.3347 | 138.169 | **<2e-16** |
|  |  |  | Surface-Ventral | -5.25 | 6.1301 | -0.856 | 0.40 |
|  |  |  | Sex-Female | -0.4167 | 7.5079 | -0.055 | 0.96 |
|  |  |  | Surface-Ventral:Sex-female | -1.25 | 10.6177 | -0.118 | 0.91 |
|  |  | *Vanessa indica* | (Intercept) | 573.75 | 7.85 | 73.091 | **<2e-16** |
|  |  |  | Surface-Ventral | -4.083 | 13.596 | -0.3 | 0.77 |
|  |  |  | Sex-Female | 5.75 | 11.101 | 0.518 | 0.61 |
|  |  |  | Surface-Ventral:Sex-female | 34.083 | 19.228 | 1.773 | 0.09 |
|  | S8 | *Papilio clytia* | (Intercept) | 2.86097 | 0.10167 | 28.14 | **<2e-16** |
|  |  |  | Surface-Ventral | 0.19018 | 0.14378 | 1.323 | 0.19 |
|  |  |  | Sex-Female | 0.07827 | 0.14378 | 0.544 | 0.59 |
|  |  |  | Surface-Ventral:Sex-female | -0.26935 | 0.20334 | -1.325 | 0.19 |
|  |  | *Doleschallia bisaltide* | (Intercept) | 2.79006 | 0.07766 | 35.926 | **< 2e-16** |
|  |  |  | Surface-Ventral | -0.66525 | 0.09512 | -6.994 | **0.00** |
|  |  |  | Sex-Female | -0.36852 | 0.12682 | -2.906 | **0.01** |
|  |  |  | Surface-Ventral:Sex-female | 0.54242 | 0.15532 | 3.492 | **0.00** |
|  |  | *Elymnias caudata* | (Intercept) | 3.536 | 0.1446 | 24.457 | **< 2e-16** |
|  |  |  | Surface-Ventral | -0.7344 | 0.1866 | -3.935 | **0.00** |
|  |  |  | Sex-Female | -0.8771 | 0.2045 | -4.29 | **0.00** |
|  |  |  | Surface-Ventral:Sex-female | 0.1677 | 0.264 | 0.635 | 0.53 |
|  |  | *Hypolimnas bolina* | (Intercept) | 2.87431 | 0.08839 | 32.518 | **<2e-16** |
|  |  |  | Surface-Ventral | -0.31829 | 0.12501 | -2.546 | **0.01** |
|  |  |  | Sex-Female | 0.2051 | 0.13694 | 1.498 | 0.14 |
|  |  |  | Surface-Ventral:Sex-female | -0.06859 | 0.19366 | -0.354 | 0.72 |
|  |  | *Melanitis leda* | (Intercept) | 2.33694 | 0.09489 | 24.629 | **<2e-16** |
|  |  |  | Surface-Ventral | 0.01756 | 0.13419 | 0.131 | 0.90 |
|  |  |  | Sex-Female | -0.01063 | 0.16435 | -0.065 | 0.95 |
|  |  |  | Surface-Ventral:Sex-female | -0.19216 | 0.23243 | -0.827 | 0.41 |
|  |  | *Vanessa indica* | (Intercept) | 2.34506 | 0.09948 | 23.573 | **<2e-16** |
|  |  |  | Surface-Ventral | -0.15248 | 0.17231 | -0.885 | 0.38 |
|  |  |  | Sex-Female | -0.15874 | 0.14069 | -1.128 | 0.27 |
|  |  |  | Surface-Ventral:Sex-female | 0.12621 | 0.24368 | 0.518 | 0.61 |
| Red-Orange | B1 | *Pachliopta aristolochiae* | (Intercept) | 2785.6 | 693 | 4.02 | **0.00** |
|  |  |  | Surface-Ventral | 3560.6 | 980 | 3.633 | **0.00** |
|  |  |  | Sex-Female | -788.7 | 1131.7 | -0.697 | 0.50 |
|  |  |  | Surface-Ventral:Sex-female | 1354.3 | 1600.4 | 0.846 | 0.41 |
|  |  | *Byasa latreillei* | (Intercept) | 4437.4 | 321.4 | 13.807 | **0.00** |
|  |  |  | Surface-Ventral | 3405.5 | 454.5 | 7.492 | **0.00** |
|  |  |  | Sex-Female | 1271.7 | 431.2 | 2.949 | **0.01** |
|  |  |  | Surface-Ventral:Sex-female | -2490 | 609.8 | -4.083 | **0.00** |
|  |  | *Cethosia biblis* | (Intercept) | 6317.3 | 279.1 | 22.632 | **< 2e-16** |
|  |  |  | Surface-Ventral | -1633.9 | 483.5 | -3.379 | **0.00** |
|  |  |  | Sex-Female | -919.2 | 455.8 | -2.017 | 0.06 |
|  |  |  | Surface-Ventral:Sex-female | 552.5 | 702.5 | 0.786 | 0.44 |
|  |  | *Papilio demoleus* | (Intercept) | 2600.76 | 161.11 | 16.143 | **<2e-16** |
|  |  |  | Surface-Ventral | 4391.05 | 227.84 | 19.272 | **<2e-16** |
|  |  |  | Sex-Female | -98.21 | 227.84 | -0.431 | 0.67 |
|  |  |  | Surface-Ventral:Sex-female | 230.4 | 322.22 | 0.715 | 0.48 |
|  |  | *Papilio helenus* | (Intercept) | 1022.62 | 185.99 | 5.498 | **0.00** |
|  |  |  | Surface-Ventral | 2425.58 | 246.05 | 9.858 | **0.00** |
|  |  |  | Sex-Female | 481.26 | 294.08 | 1.636 | 0.12 |
|  |  |  | Surface-Ventral:Sex-female | -28.62 | 405.36 | -0.071 | 0.95 |
|  |  | *Vanessa indica* | (Intercept) | 4601.4 | 219.2 | 20.995 | **< 2e-16** |
|  |  |  | Surface-Ventral | 1900.6 | 379.6 | 5.007 | **0.00** |
|  |  |  | Sex-Female | 741.5 | 310 | 2.392 | **0.02** |
|  |  |  | Surface-Ventral:Sex-female | -346 | 536.9 | -0.645 | 0.52 |
|  | H3 | *Pachliopta aristolochiae* | (Intercept) | 608.8 | 1.893 | 321.661 | **<2e-16** |
|  |  |  | Surface-Ventral | -1.6 | 2.677 | -0.598 | 0.56 |
|  |  |  | Sex-Female | -1.467 | 3.091 | -0.475 | 0.64 |
|  |  |  | Surface-Ventral:Sex-female | -1.067 | 4.371 | -0.244 | 0.81 |
|  |  | *Byasa latreillei* | (Intercept) | 592.5 | 2.237 | 264.903 | **< 2e-16** |
|  |  |  | Surface-Ventral | 2.25 | 3.163 | 0.711 | 0.49 |
|  |  |  | Sex-Female | 11.1 | 3.001 | 3.699 | **0.00** |
|  |  |  | Surface-Ventral:Sex-female | 0.55 | 4.244 | 0.13 | 0.90 |
|  |  | *Cethosia biblis* | (Intercept) | 463.9 | 38.708 | 11.985 | **0.00** |
|  |  |  | Surface-Ventral | -12.5 | 67.045 | -0.186 | 0.85 |
|  |  |  | Sex-Female | 57.1 | 63.21 | 0.903 | 0.38 |
|  |  |  | Surface-Ventral:Sex-female | 3.333 | 97.414 | 0.034 | 0.97 |
|  |  | *Papilio demoleus* | (Intercept) | 637.3 | 1.588 | 401.24 | **<2e-16** |
|  |  |  | Surface-Ventral | -81 | 2.246 | -36.06 | **<2e-16** |
|  |  |  | Sex-Female | 2.5 | 2.246 | 1.113 | 0.27 |
|  |  |  | Surface-Ventral:Sex-female | -6.2 | 3.177 | -1.952 | 0.06 |
|  |  | *Papilio helenus* | (Intercept) | 645.3333 | 4.3864 | 147.122 | **< 2e-16** |
|  |  |  | Surface-Ventral | -31.9583 | 5.8026 | -5.508 | **0.00** |
|  |  |  | Sex-Female | 0.6667 | 6.9355 | 0.096 | 0.92 |
|  |  |  | Surface-Ventral:Sex-female | 8.7083 | 9.5599 | 0.911 | 0.37 |
|  |  | *Vanessa indica* | (Intercept) | 573.1667 | 1.7356 | 330.25 | **<2e-16** |
|  |  |  | Surface-Ventral | -0.1667 | 3.0061 | -0.055 | 0.96 |
|  |  |  | Sex-Female | 3.3333 | 2.4544 | 1.358 | 0.18 |
|  |  |  | Surface-Ventral:Sex-female | -4.3333 | 4.2512 | -1.019 | 0.32 |
|  | S8 | *Pachliopta aristolochiae* | (Intercept) | 2.91819 | 0.0711 | 41.042 | **0.00** |
|  |  |  | Surface-Ventral | 0.52979 | 0.10055 | 5.269 | **0.00** |
|  |  |  | Sex-Female | -0.205 | 0.11611 | -1.766 | 0.10 |
|  |  |  | Surface-Ventral:Sex-female | 0.02946 | 0.1642 | 0.179 | 0.86 |
|  |  | *Byasa latreillei* | (Intercept) | 2.51786 | 0.11897 | 21.164 | **0.00** |
|  |  |  | Surface-Ventral | 0.27458 | 0.16825 | 1.632 | 0.12 |
|  |  |  | Sex-Female | 0.6261 | 0.15961 | 3.923 | **0.00** |
|  |  |  | Surface-Ventral:Sex-female | -0.08459 | 0.22573 | -0.375 | 0.71 |
|  |  | *Cethosia biblis* | (Intercept) | 2.3581 | 0.08496 | 27.755 | **< 2e-16** |
|  |  |  | Surface-Ventral | 0.05029 | 0.14716 | 0.342 | 0.74 |
|  |  |  | Sex-Female | -0.47019 | 0.13874 | -3.389 | **0.00** |
|  |  |  | Surface-Ventral:Sex-female | 0.35561 | 0.21381 | 1.663 | 0.11 |
|  |  | *Papilio demoleus* | (Intercept) | 5.10106 | 0.07049 | 72.362 | **<2e-16** |
|  |  |  | Surface-Ventral | -2.38116 | 0.09969 | -23.885 | **<2e-16** |
|  |  |  | Sex-Female | 0.05455 | 0.09969 | 0.547 | 0.59 |
|  |  |  | Surface-Ventral:Sex-female | -0.1903 | 0.14099 | -1.35 | 0.19 |
|  |  | *Papilio helenus* | (Intercept) | 5.25926 | 0.1997 | 26.336 | **0.00** |
|  |  |  | Surface-Ventral | -1.51027 | 0.26418 | -5.717 | **0.00** |
|  |  |  | Sex-Female | -0.11291 | 0.31576 | -0.358 | 0.73 |
|  |  |  | Surface-Ventral:Sex-female | 0.07782 | 0.43524 | 0.179 | 0.86 |
|  |  | *Vanessa indica* | (Intercept) | 2.8606 | 0.07815 | 36.605 | **< 2e-16** |
|  |  |  | Surface-Ventral | -0.46502 | 0.13536 | -3.436 | **0.00** |
|  |  |  | Sex-Female | 0.01567 | 0.11052 | 0.142 | 0.89 |
|  |  |  | Surface-Ventral:Sex-female | -0.05811 | 0.19142 | -0.304 | 0.76 |
|  | S1R | *Pachliopta aristolochiae* | (Intercept) | 0.61575 | 0.009627 | 63.96 | **< 2e-16** |
|  |  |  | Surface-Ventral | 0.100454 | 0.013615 | 7.378 | **0.00** |
|  |  |  | Sex-Female | -0.03813 | 0.015721 | -2.425 | **0.03** |
|  |  |  | Surface-Ventral:Sex-female | 0.016176 | 0.022233 | 0.728 | 0.48 |
|  |  | *Byasa latreillei* | (Intercept) | 0.57388 | 0.01753 | 32.731 | **0.00** |
|  |  |  | Surface-Ventral | 0.05766 | 0.0248 | 2.325 | **0.04** |
|  |  |  | Sex-Female | 0.10291 | 0.02352 | 4.375 | **0.00** |
|  |  |  | Surface-Ventral:Sex-female | -0.02974 | 0.03327 | -0.894 | 0.39 |
|  |  | *Cethosia biblis* | (Intercept) | 0.52576 | 0.01573 | 33.428 | **<2e-16** |
|  |  |  | Surface-Ventral | -0.01453 | 0.02724 | -0.533 | 0.60 |
|  |  |  | Sex-Female | -0.0492 | 0.02568 | -1.916 | 0.07 |
|  |  |  | Surface-Ventral:Sex-female | 0.02351 | 0.03958 | 0.594 | 0.56 |
|  |  | *Papilio demoleus* | (Intercept) | 0.787543 | 0.007083 | 111.192 | **<2e-16** |
|  |  |  | Surface-Ventral | -0.20086 | 0.010017 | -20.053 | **<2e-16** |
|  |  |  | Sex-Female | -0.00663 | 0.010017 | -0.662 | 0.51 |
|  |  |  | Surface-Ventral:Sex-female | -0.00967 | 0.014165 | -0.683 | 0.50 |
|  |  | *Papilio helenus* | (Intercept) | 0.76097 | 0.01879 | 40.506 | **<2e-16** |
|  |  |  | Surface-Ventral | -0.07044 | 0.02485 | -2.834 | **0.01** |
|  |  |  | Sex-Female | -0.01295 | 0.0297 | -0.436 | 0.67 |
|  |  |  | Surface-Ventral:Sex-female | -0.00321 | 0.04095 | -0.078 | 0.94 |
|  |  | *Vanessa indica* | (Intercept) | 0.648879 | 0.009363 | 69.303 | **< 2e-16** |
|  |  |  | Surface-Ventral | -0.08468 | 0.016217 | -5.222 | **0.00** |
|  |  |  | Sex-Female | -0.00962 | 0.013241 | -0.726 | 0.47 |
|  |  |  | Surface-Ventral:Sex-female | 0.00529 | 0.022934 | 0.231 | 0.82 |
| White | B1 | *Pachliopta aristolochiae* | (Intercept) | 12632 | 473.4 | 26.683 | **0.00** |
|  |  |  | Surface-Ventral | 592.1 | 669.5 | 0.884 | 0.39 |
|  |  |  | Sex-Female | -1004.5 | 773.1 | -1.299 | 0.22 |
|  |  |  | Surface-Ventral:Sex-female | 839 | 1093.3 | 0.767 | 0.46 |
|  |  | *Byasa latreillei* | (Intercept) | 11403.19 | 876.2 | 13.014 | **0.00** |
|  |  |  | Surface-Ventral | 33.37 | 1239.13 | 0.027 | 0.98 |
|  |  |  | Sex-Female | 1399.65 | 1175.55 | 1.191 | 0.25 |
|  |  |  | Surface-Ventral:Sex-female | -1240.26 | 1662.47 | -0.746 | 0.47 |
|  |  | *Cethosia biblis* | (Intercept) | 14516.8 | 710.4 | 20.434 | **< 2e-16** |
|  |  |  | Surface-Ventral | -3938.4 | 870.1 | -4.527 | **0.00** |
|  |  |  | Sex-Female | -622.1 | 961.9 | -0.647 | 0.52 |
|  |  |  | Surface-Ventral:Sex-female | 479 | 1178.1 | 0.407 | 0.69 |
|  |  | *Papilio clytia* | (Intercept) | 9655.7 | 856.6 | 11.272 | **0.00** |
|  |  |  | Surface-Ventral | 4859.9 | 1211.4 | 4.012 | **0.00** |
|  |  |  | Sex-Female | 1271.3 | 1211.4 | 1.049 | 0.31 |
|  |  |  | Surface-Ventral:Sex-female | -1751.6 | 1713.2 | -1.022 | 0.32 |
|  |  | *Colotis danae* | (Intercept) | 17761 | 520.38 | 34.131 | **< 2e-16** |
|  |  |  | Surface-Ventral | -89.43 | 735.92 | -0.122 | 0.90 |
|  |  |  | Sex-Female | -3429.17 | 735.92 | -4.66 | **0.00** |
|  |  |  | Surface-Ventral:Sex-female | 884.91 | 1040.75 | 0.85 | 0.40 |
|  |  | *Delias descombesi* | (Intercept) | 21045.2 | 423.3 | 49.722 | **< 2e-16** |
|  |  |  | Surface-Ventral | -237.4 | 733.1 | -0.324 | 0.75 |
|  |  |  | Sex-Female | -4983.8 | 598.6 | -8.326 | **0.00** |
|  |  |  | Surface-Ventral:Sex-female | 2183 | 1036.8 | 2.106 | **0.04** |
|  |  | *Elymnias caudata* | (Intercept) | 15473.9 | 420.8 | 36.77 | **<2e-16** |
|  |  |  | Surface-Ventral | -9019.3 | 595.1 | -15.155 | **<2e-16** |
|  |  |  | Sex-Female | 64.9 | 595.1 | 0.109 | 0.91 |
|  |  |  | Surface-Ventral:Sex-female | 1251.2 | 841.7 | 1.487 | 0.15 |
|  |  | *Hebomoia glaucippe* | (Intercept) | 20397.1 | 418.9 | 48.697 | **< 2e-16** |
|  |  |  | Surface-Ventral | -1860.5 | 725.5 | -2.564 | **0.02** |
|  |  |  | Sex-Female | -6305.1 | 662.3 | -9.52 | **0.00** |
|  |  |  | Surface-Ventral:Sex-female | 588.7 | 1147.1 | 0.513 | 0.61 |
|  |  | *Papilio helenus* | (Intercept) | 19427.1 | 390.4 | 49.764 | **< 2e-16** |
|  |  |  | Surface-Ventral | 346.6 | 552.1 | 0.628 | 0.54 |
|  |  |  | Sex-Female | -3163.5 | 676.2 | -4.679 | **0.00** |
|  |  |  | Surface-Ventral:Sex-female | 1906.9 | 956.2 | 1.994 | 0.06 |
|  |  | *Hypolimnas bolina* | (Intercept) | 14698 | 783.3 | 18.764 | **< 2e-16** |
|  |  |  | Surface-Ventral | -3066.7 | 1107.7 | -2.768 | **0.01** |
|  |  |  | Sex-Female | -1120.8 | 1213.5 | -0.924 | 0.36 |
|  |  |  | Surface-Ventral:Sex-female | 1452.9 | 1716.1 | 0.847 | 0.40 |
|  |  | *Pareronia hippia* | (Intercept) | 14052.9 | 573.1 | 24.523 | **< 2e-16** |
|  |  |  | Surface-Ventral | 254.3 | 467.9 | 0.543 | 0.59 |
|  |  |  | Sex-Female | -1987.3 | 467.9 | -4.247 | **0.00** |
|  |  | *Pieris canidia* | (Intercept) | 17338.6 | 516.8 | 33.55 | **< 2e-16** |
|  |  |  | Surface-Ventral | -4357.6 | 730.9 | -5.962 | **0.00** |
|  |  |  | Sex-Female | -2498.8 | 730.9 | -3.419 | **0.00** |
|  |  |  | Surface-Ventral:Sex-female | 1753.1 | 1033.6 | 1.696 | 0.09 |
|  |  | *Vanessa indica* | (Intercept) | 13296.6 | 659.7 | 20.156 | **0.00** |
|  |  |  | Surface-Ventral | 604 | 932.9 | 0.647 | 0.53 |
|  |  |  | Sex-Female | 649.3 | 932.9 | 0.696 | 0.49 |
|  |  |  | Surface-Ventral:Sex-female | 645.6 | 1319.4 | 0.489 | 0.63 |
|  | H3 | *Pachliopta aristolochiae* | (Intercept) | 501.2 | 5.617 | 89.232 | **<2e-16** |
|  |  |  | Surface-Ventral | -11.4 | 7.943 | -1.435 | 0.18 |
|  |  |  | Sex-Female | -8.533 | 9.172 | -0.93 | 0.37 |
|  |  |  | Surface-Ventral:Sex-female | 2.067 | 12.972 | 0.159 | 0.88 |
|  |  | *Byasa latreillei* | (Intercept) | 439.25 | 10.88 | 40.373 | **0.00** |
|  |  |  | Surface-Ventral | 7.75 | 15.39 | 0.504 | 0.62 |
|  |  |  | Sex-Female | -6.05 | 14.6 | -0.414 | 0.69 |
|  |  |  | Surface-Ventral:Sex-female | 10.65 | 20.64 | 0.516 | 0.61 |
|  |  | *Cethosia biblis* | (Intercept) | 366 | 12.57 | 29.11 | **< 2e-16** |
|  |  |  | Surface-Ventral | 128.4 | 15.4 | 8.338 | **0.00** |
|  |  |  | Sex-Female | 20.17 | 17.02 | 1.185 | 0.25 |
|  |  |  | Surface-Ventral:Sex-female | -63.48 | 20.85 | -3.045 | **0.00** |
|  |  | *Papilio clytia* | (Intercept) | 465.667 | 9.424 | 49.411 | **<2e-16** |
|  |  |  | Surface-Ventral | -36.667 | 13.328 | -2.751 | **0.01** |
|  |  |  | Sex-Female | -23 | 13.328 | -1.726 | 0.10 |
|  |  |  | Surface-Ventral:Sex-female | 18.667 | 18.849 | 0.99 | 0.33 |
|  |  | *Colotis danae* | (Intercept) | 425.8 | 1.029 | 413.79 | **< 2e-16** |
|  |  |  | Surface-Ventral | -2.6 | 1.455 | -1.787 | 0.08 |
|  |  |  | Sex-Female | -4.6 | 1.455 | -3.161 | **0.00** |
|  |  |  | Surface-Ventral:Sex-female | 6 | 2.058 | 2.915 | **0.01** |
|  |  | *Delias descombesi* | (Intercept) | 424.25 | 7.876 | 53.866 | **<2e-16** |
|  |  |  | Surface-Ventral | -0.95 | 13.642 | -0.07 | 0.95 |
|  |  |  | Sex-Female | -13.15 | 11.138 | -1.181 | 0.24 |
|  |  |  | Surface-Ventral:Sex-female | -23.85 | 19.292 | -1.236 | 0.22 |
|  |  | *Elymnias caudata* | (Intercept) | 355 | 8.181 | 43.392 | **<2e-16** |
|  |  |  | Surface-Ventral | 222.9 | 11.57 | 19.265 | **<2e-16** |
|  |  |  | Sex-Female | 6.6 | 11.57 | 0.57 | 0.57 |
|  |  |  | Surface-Ventral:Sex-female | -43.4 | 16.363 | -2.652 | **0.01** |
|  |  | *Hebomoia glaucippe* | (Intercept) | 428.25 | 0.7059 | 606.691 | **< 2e-16** |
|  |  |  | Surface-Ventral | -2.9167 | 1.2226 | -2.386 | **0.02** |
|  |  |  | Sex-Female | 4.875 | 1.1161 | 4.368 | **0.00** |
|  |  |  | Surface-Ventral:Sex-female | -3.7083 | 1.9331 | -1.918 | 0.07 |
|  |  | *Papilio helenus* | (Intercept) | 441.25 | 1.557 | 283.308 | **< 2e-16** |
|  |  |  | Surface-Ventral | -14.625 | 2.203 | -6.64 | **0.00** |
|  |  |  | Sex-Female | -1.5 | 2.698 | -0.556 | 0.58 |
|  |  |  | Surface-Ventral:Sex-female | -7.125 | 3.815 | -1.868 | 0.08 |
|  |  | *Hypolimnas bolina* | (Intercept) | 453.357 | 24.144 | 18.778 | **<2e-16** |
|  |  |  | Surface-Ventral | 3.643 | 34.144 | 0.107 | 0.92 |
|  |  |  | Sex-Female | 40.643 | 37.403 | 1.087 | 0.28 |
|  |  |  | Surface-Ventral:Sex-female | -5.443 | 52.896 | -0.103 | 0.92 |
|  |  | *Pareronia hippia* | (Intercept) | 372.55 | 10.894 | 34.197 | **< 2e-16** |
|  |  |  | Surface-Ventral | 4.3 | 8.895 | 0.483 | 0.63 |
|  |  |  | Sex-Female | 48.65 | 8.895 | 5.469 | **0.00** |
|  |  | *Pieris canidia* | (Intercept) | 425.6 | 2.574 | 165.348 | **<2e-16** |
|  |  |  | Surface-Ventral | -0.3 | 3.64 | -0.082 | 0.93 |
|  |  |  | Sex-Female | -3.15 | 3.64 | -0.865 | 0.39 |
|  |  |  | Surface-Ventral:Sex-female | 14 | 5.148 | 2.72 | **0.01** |
|  |  | *Vanessa indica* | (Intercept) | 460.167 | 25.054 | 18.367 | **0.00** |
|  |  |  | Surface-Ventral | 23.833 | 35.432 | 0.673 | 0.51 |
|  |  |  | Sex-Female | -1.833 | 35.432 | -0.052 | 0.96 |
|  |  |  | Surface-Ventral:Sex-female | -76 | 50.108 | -1.517 | 0.15 |
|  | S8 | *Pachliopta aristolochiae* | (Intercept) | 1.56243 | 0.04036 | 38.713 | **0.00** |
|  |  |  | Surface-Ventral | -0.1873 | 0.05708 | -3.282 | **0.01** |
|  |  |  | Sex-Female | -0.04359 | 0.06591 | -0.661 | 0.52 |
|  |  |  | Surface-Ventral:Sex-female | 0.08289 | 0.09321 | 0.889 | 0.39 |
|  |  | *Byasa latreillei* | (Intercept) | 1.06291 | 0.05232 | 20.317 | **0.00** |
|  |  |  | Surface-Ventral | -0.02156 | 0.07399 | -0.291 | 0.78 |
|  |  |  | Sex-Female | -0.15211 | 0.07019 | -2.167 | **0.05** |
|  |  |  | Surface-Ventral:Sex-female | 0.05205 | 0.09926 | 0.524 | 0.61 |
|  |  | *Cethosia biblis* | (Intercept) | 0.580797 | 0.069476 | 8.36 | **0.00** |
|  |  |  | Surface-Ventral | 0.673419 | 0.08509 | 7.914 | **0.00** |
|  |  |  | Sex-Female | -0.00343 | 0.094071 | -0.036 | 0.97 |
|  |  |  | Surface-Ventral:Sex-female | -0.24706 | 0.115213 | -2.144 | **0.04** |
|  |  | *Papilio clytia* | (Intercept) | 1.52458 | 0.06513 | 23.408 | **0.00** |
|  |  |  | Surface-Ventral | -0.39584 | 0.09211 | -4.298 | **0.00** |
|  |  |  | Sex-Female | -0.17668 | 0.09211 | -1.918 | 0.07 |
|  |  |  | Surface-Ventral:Sex-female | 0.17884 | 0.13026 | 1.373 | 0.18 |
|  |  | *Colotis danae* | (Intercept) | 1.43882 | 0.02154 | 66.797 | **< 2e-16** |
|  |  |  | Surface-Ventral | -0.03572 | 0.03046 | -1.173 | 0.25 |
|  |  |  | Sex-Female | -0.147 | 0.03046 | -4.825 | **0.00** |
|  |  |  | Surface-Ventral:Sex-female | 0.12571 | 0.04308 | 2.918 | **0.01** |
|  |  | *Delias descombesi* | (Intercept) | 1.4147 | 0.0256 | 55.254 | **< 2e-16** |
|  |  |  | Surface-Ventral | -0.0382 | 0.04435 | -0.861 | 0.39 |
|  |  |  | Sex-Female | -0.32155 | 0.03621 | -8.88 | **0.00** |
|  |  |  | Surface-Ventral:Sex-female | -0.01452 | 0.06272 | -0.232 | 0.82 |
|  |  | *Elymnias caudata* | (Intercept) | 0.75227 | 0.04912 | 15.314 | **< 2e-16** |
|  |  |  | Surface-Ventral | 0.52401 | 0.06947 | 7.543 | **0.00** |
|  |  |  | Sex-Female | -0.01849 | 0.06947 | -0.266 | 0.79 |
|  |  |  | Surface-Ventral:Sex-female | -0.11645 | 0.09825 | -1.185 | 0.24 |
|  |  | *Hebomoia glaucippe* | (Intercept) | 1.444018 | 0.013921 | 103.733 | **< 2e-16** |
|  |  |  | Surface-Ventral | -0.08017 | 0.024111 | -3.325 | **0.00** |
|  |  |  | Sex-Female | -0.03087 | 0.02201 | -1.403 | 0.17 |
|  |  |  | Surface-Ventral:Sex-female | 0.007515 | 0.038123 | 0.197 | 0.85 |
|  |  | *Papilio helenus* | (Intercept) | 1.5206 | 0.01218 | 124.797 | **< 2e-16** |
|  |  |  | Surface-Ventral | -0.4854 | 0.01723 | -28.169 | **< 2e-16** |
|  |  |  | Sex-Female | -0.04393 | 0.0211 | -2.082 | 0.05 |
|  |  |  | Surface-Ventral:Sex-female | -0.11182 | 0.02985 | -3.747 | **0.00** |
|  |  | *Hypolimnas bolina* | (Intercept) | 0.65886 | 0.05754 | 11.45 | **0.00** |
|  |  |  | Surface-Ventral | 0.13556 | 0.08138 | 1.666 | 0.10 |
|  |  |  | Sex-Female | 0.57106 | 0.08915 | 6.406 | **0.00** |
|  |  |  | Surface-Ventral:Sex-female | -0.12663 | 0.12607 | -1.004 | 0.32 |
|  |  | *Pareronia hippia* | (Intercept) | 1.34521 | 0.06568 | 20.481 | **< 2e-16** |
|  |  |  | Surface-Ventral | -0.19767 | 0.05363 | -3.686 | **0.00** |
|  |  |  | Sex-Female | 0.09293 | 0.05363 | 1.733 | 0.09 |
|  |  | *Pieris canidia* | (Intercept) | 1.44274 | 0.01076 | 134.033 | **< 2e-16** |
|  |  |  | Surface-Ventral | -0.05142 | 0.01522 | -3.378 | **0.00** |
|  |  |  | Sex-Female | -0.08419 | 0.01522 | -5.53 | **0.00** |
|  |  |  | Surface-Ventral:Sex-female | 0.08714 | 0.02153 | 4.048 | **0.00** |
|  |  | *Vanessa indica* | (Intercept) | 1.0814 | 0.06189 | 17.472 | **0.00** |
|  |  |  | Surface-Ventral | -0.11918 | 0.08753 | -1.362 | 0.19 |
|  |  |  | Sex-Female | -0.07629 | 0.08753 | -0.872 | 0.39 |
|  |  |  | Surface-Ventral:Sex-female | -0.06801 | 0.12379 | -0.549 | 0.59 |
| Yellow | B1 | *Colias fieldii* | (Intercept) | 9339.1 | 431.1 | 21.664 | **< 2e-16** |
|  |  |  | Surface-Ventral | 605.9 | 528 | 1.148 | 0.26 |
|  |  |  | Sex-Female | 1798 | 609.6 | 2.949 | **0.01** |
|  |  |  | Surface-Ventral:Sex-female | -3306.5 | 746.7 | -4.428 | **0.00** |
|  |  | *Papilio demoleus* | (Intercept) | 17612.86 | 274.9 | 64.07 | **<2e-16** |
|  |  |  | Surface-Ventral | -79.25 | 388.77 | -0.204 | 0.84 |
|  |  |  | Sex-Female | -926.42 | 388.77 | -2.383 | **0.02** |
|  |  |  | Surface-Ventral:Sex-female | 208.63 | 549.8 | 0.379 | 0.71 |
|  |  | *Ixias pyrene* | (Intercept) | 16515.6 | 263.9 | 62.581 | **< 2e-16** |
|  |  |  | Surface-Ventral | -1018.3 | 373.2 | -2.729 | **0.01** |
|  |  |  | Sex-Female | -3204.4 | 373.2 | -8.586 | **0.00** |
|  |  | *Junonia hierta* | (Intercept) | 11502.9 | 379.2 | 30.336 | **<2e-16** |
|  |  |  | Surface-Ventral | 1538.4 | 656.8 | 2.342 | **0.02** |
|  |  |  | Sex-Female | -876.1 | 590.9 | -1.483 | 0.14 |
|  |  |  | Surface-Ventral:Sex-female | -1457.7 | 1023.5 | -1.424 | 0.16 |
|  | H3 | *Colias fieldii* | (Intercept) | 467 | 6.96 | 67.097 | **< 2e-16** |
|  |  |  | Surface-Ventral | 44.786 | 8.524 | 5.254 | **0.00** |
|  |  |  | Sex-Female | 1.429 | 9.843 | 0.145 | 0.89 |
|  |  |  | Surface-Ventral:Sex-female | -2.714 | 12.055 | -0.225 | 0.82 |
|  |  | *Papilio demoleus* | (Intercept) | 447.65 | 0.6792 | 659.058 | **< 2e-16** |
|  |  |  | Surface-Ventral | -6.25 | 0.9606 | -6.507 | **0.00** |
|  |  |  | Sex-Female | 5.9 | 0.9606 | 6.142 | **0.00** |
|  |  |  | Surface-Ventral:Sex-female | -1.25 | 1.3585 | -0.92 | 0.36 |
|  |  | *Ixias pyrene* | (Intercept) | 481.75 | 3.103 | 155.259 | **<2e-16** |
|  |  |  | Surface-Ventral | 6.8 | 4.388 | 1.55 | 0.13 |
|  |  |  | Sex-Female | -3.05 | 4.388 | -0.695 | 0.49 |
|  |  | *Junonia hierta* | (Intercept) | 515.4 | 2.893 | 178.184 | **<2e-16** |
|  |  |  | Surface-Ventral | 3.4 | 5.01 | 0.679 | 0.50 |
|  |  |  | Sex-Female | 4.886 | 4.508 | 1.084 | 0.28 |
|  |  |  | Surface-Ventral:Sex-female | 14.6 | 7.808 | 1.87 | 0.07 |
|  | S8 | *Colias fieldii* | (Intercept) | 1.32011 | 0.07826 | 16.868 | **< 2e-16** |
|  |  |  | Surface-Ventral | 0.54757 | 0.09585 | 5.713 | **0.00** |
|  |  |  | Sex-Female | 0.05552 | 0.11068 | 0.502 | 0.62 |
|  |  |  | Surface-Ventral:Sex-female | -0.12251 | 0.13555 | -0.904 | 0.37 |
|  |  | *Papilio demoleus* | (Intercept) | 1.603656 | 0.007668 | 209.125 | **< 2e-16** |
|  |  |  | Surface-Ventral | -0.12045 | 0.010845 | -11.107 | **< 2e-16** |
|  |  |  | Sex-Female | 0.050709 | 0.010845 | 4.676 | **0.00** |
|  |  |  | Surface-Ventral:Sex-female | -0.02045 | 0.015337 | -1.334 | 0.19 |
|  |  | *Ixias pyrene* | (Intercept) | 1.79863 | 0.02399 | 74.983 | **< 2e-16** |
|  |  |  | Surface-Ventral | -0.04864 | 0.03392 | -1.434 | 0.16 |
|  |  |  | Sex-Female | -0.09194 | 0.03392 | -2.71 | **0.01** |
|  |  | *Junonia hierta* | (Intercept) | 2.22003 | 0.03306 | 67.155 | **<2e-16** |
|  |  |  | Surface-Ventral | -0.11554 | 0.05726 | -2.018 | **0.05** |
|  |  |  | Sex-Female | -0.04637 | 0.05152 | -0.9 | 0.37 |
|  |  |  | Surface-Ventral:Sex-female | 0.15716 | 0.08923 | 1.761 | 0.08 |
|  | S1Y | *Colias fieldii* | (Intercept) | 0.285113 | 0.00986 | 28.917 | **< 2e-16** |
|  |  |  | Surface-Ventral | 0.061938 | 0.012076 | 5.129 | **0.00** |
|  |  |  | Sex-Female | -0.00361 | 0.013944 | -0.259 | 0.80 |
|  |  |  | Surface-Ventral:Sex-female | -0.00846 | 0.017078 | -0.495 | 0.62 |
|  |  | *Papilio demoleus* | (Intercept) | 0.29454 | 0.001149 | 256.321 | **< 2e-16** |
|  |  |  | Surface-Ventral | -0.01397 | 0.001625 | -8.593 | **0.00** |
|  |  |  | Sex-Female | 0.01376 | 0.001625 | 8.467 | **0.00** |
|  |  |  | Surface-Ventral:Sex-female | -0.00677 | 0.002298 | -2.946 | **0.00** |
|  |  | *Ixias pyrene* | (Intercept) | 0.340523 | 0.003343 | 101.873 | **< 2e-16** |
|  |  |  | Surface-Ventral | -0.00393 | 0.004727 | -0.83 | 0.41 |
|  |  |  | Sex-Female | -0.0183 | 0.004727 | -3.871 | **0.00** |
|  |  | *Junonia hierta* | (Intercept) | 0.336175 | 0.001197 | 280.784 | **< 2e-16** |
|  |  |  | Surface-Ventral | -0.02331 | 0.002074 | -11.241 | **0.00** |
|  |  |  | Sex-Female | -0.00344 | 0.001866 | -1.841 | 0.07 |
|  |  |  | Surface-Ventral:Sex-female | 0.005095 | 0.003232 | 1.576 | 0.12 |

**Table S4:** T-tests (t-statistic) or Wilcoxon rank-sum tests (W) for spectral colour parameters for each species separated by colour. Spectral colour parameters: Brightness (B1), Hue (H3, H1), and Saturation (S8, S1R, S1Y, S1B). Significant values are in bold. These results correspond to Fig. 1a–f.

| **Colour** | **Colour parameter** | **Species** | **t-statistic** | **W** | **p-value** |
| --- | --- | --- | --- | --- | --- |
| Black | B1 | *Colotis danae* | 0.037 |  | 0.97 |
|  |  | *Doleschallia bisaltidae* | -1.592 |  | 0.20 |
|  |  | *Melanitis leda* | 0.346 |  | 0.74 |
|  |  | *Cethosia biblis* | | 33.000 | 0.08 |
|  |  | *Ixias pyrene* | | 33.000 | **0.00** |
|  |  | *Junonia hierta* | | 9.000 | **0.00** |
|  |  | *Tajuria cippus* | | 1.000 | **0.00** |
|  | H3 | *Colotis danae* | -1.37611 |  | 0.19 |
|  |  | *Doleschallia bisaltidae* | 2.193492 |  | 0.07 |
|  |  | *Ixias pyrene* | 0.045514 |  | 0.96 |
|  |  | *Junonia hierta* | 1.506525 |  | 0.14 |
|  |  | *Tajuria cippus* | 1.601366 |  | 0.13 |
|  |  | *Cethosia biblis* | | 58.5 | 0.95 |
|  |  | *Melanitis leda* | | 7 | 0.69 |
|  | S8 | *Cethosia biblis* | -1.24 |  | 0.23 |
|  |  | *Colotis danae* | -0.94 |  | 0.36 |
|  |  | *Doleschallia bisaltidae* | 0.14 |  | 0.90 |
|  |  | *Ixias pyrene* | 0.69 |  | 0.50 |
|  |  | *Junonia hierta* | 2.15 |  | **0.04** |
|  |  | *Melanitis leda* | -0.24 |  | 0.82 |
|  |  | *Tajuria cippus* | 1.87 |  | 0.08 |
| Brown | B1 | *Cethosia biblis* | 1.50 |  | 0.15 |
|  |  | *Colotis danae* | 0.29 |  | 0.78 |
|  |  | *Hebomoia glaucippe* | 4.51 |  | **0.00** |
|  |  | *Junonia hierta* | | 159 | 0.52 |
|  | H3 | *Cethosia biblis* | 5.78 |  | **0.00** |
|  |  | *Colotis danae* | | 8 | 0.42 |
|  |  | *Hebomoia glaucippe* | | 37 | 0.42 |
|  |  | *Junonia hierta* | | 102 | 0.19 |
|  | S8 | *Cethosia biblis* | 5.19 |  | **0.00** |
|  |  | *Colotis danae* | 0.67 |  | 0.52 |
|  |  | *Hebomoia glaucippe* | 0.19 |  | 0.85 |
|  |  | *Junonia hierta* | -0.12 |  | 0.90 |
| Red-Orange | B1 | *Colias fieldii* | 3.15 |  | **0.00** |
|  |  | *Colotis danae* | 4.42 |  | **0.01** |
|  |  | *Delias descombesi* | 2.45 |  | **0.02** |
|  |  | *Hebomoia glaucippe* | 1.83 |  | 0.11 |
|  |  | *Ixias pyrene* | | 24 | 0.05 |
|  | H3 | *Colias fieldii* | 4.16 |  | **0.00** |
|  |  | *Colotis danae* | | 10 | 0.69 |
|  |  | *Delias descombesi* | 0.32 |  | 0.75 |
|  |  | *Hebomoia glaucippe* | 3.41 |  | **0.02** |
|  |  | *Ixias pyrene* | | 40 | 0.48 |
|  | S8 | *Colias fieldii* | 0.43 |  | 0.67 |
|  |  | *Colotis danae* | -2.13 |  | 0.07 |
|  |  | *Delias descombesi* | | 61 | 0.44 |
|  |  | *Hebomoia glaucippe* | 3.82 |  | **0.02** |
|  |  | *Ixias pyrene* | 6.52 |  | **0.00** |
|  | S1R | *Colias fieldii* | 1.71 |  | 0.10 |
|  |  | *Colotis danae* | -3.55 |  | **0.01** |
|  |  | *Delias descombesi* | 0.17 |  | 0.87 |
|  |  | *Hebomoia glaucippe* | 3.49 |  | **0.02** |
|  |  | *Ixias pyrene* | | 99 | **0.00** |
| White | B1 | *Colias fieldii* | 1.59 |  | 0.14 |
|  |  | *Junonia hierta* | -0.68 |  | 0.51 |
|  |  | *Melanitis leda* | -0.98 |  | 0.37 |
|  |  | *Ixias pyrene* | | 241 | **0.00** |
|  | H3 | *Colias fieldii* | | 10.5 | 0.79 |
|  |  | *Junonia hierta* | | 16.5 | 0.34 |
|  |  | *Melanitis leda* | | 44.5 | 0.38 |
|  |  | *Ixias pyrene* | | 103.5 | 0.15 |
|  | S8 | *Colias fieldii* | -1.09 |  | 0.30 |
|  |  | *Junonia hierta* | | 33 | 0.89 |
|  |  | *Melanitis leda* | -0.20 |  | 0.85 |
|  |  | *Ixias pyrene* | | 90 | 0.06 |
| Yellow | B1 | *Cethosia biblis* | 0.16 |  | 0.88 |
|  |  | *Papilio clytia* | -2.99 |  | **0.01** |
|  |  | *Colotis danae* | -0.70 |  | 0.50 |
|  |  | *Delias descombesi* | 1.82 |  | 0.09 |
|  |  | *Elymnias caudata* | 4.01 |  | **0.00** |
|  |  | *Melanitis leda* | 0.56 |  | 0.59 |
|  | H3 | *Cethosia biblis* | | 30 | **0.01** |
|  |  | *Papilio clytia* | | 25.5 | 0.41 |
|  |  | *Colotis danae* | -1.48 |  | 0.18 |
|  |  | *Delias descombesi* | | 96.5 | **0.00** |
|  |  | *Elymnias caudata* | | 69 | 0.18 |
|  |  | *Melanitis leda* | | 6 | 0.52 |
|  | S8 | *Cethosia biblis* | 1.72 |  | 0.14 |
|  |  | *Papilio clytia* | 0.70 |  | 0.51 |
|  |  | *Colotis danae* | -0.96 |  | 0.37 |
|  |  | *Delias descombesi* | 5.27 |  | **0.00** |
|  |  | *Elymnias caudata* | | 56 | 0.05 |
|  |  | *Melanitis leda* | -0.48 |  | 0.65 |
|  | S1Y | *Cethosia biblis* | -3.03 |  | **0.02** |
|  |  | *Papilio clytia* | -0.70 |  | 0.50 |
|  |  | *Colotis danae* | -2.68 |  | **0.03** |
|  |  | *Delias descombesi* | 4.59 |  | **0.00** |
|  |  | *Elymnias caudata* | -0.97 |  | 0.35 |
|  |  | *Melanitis leda* | 0.94 |  | 0.38 |
| Blue | B1 | *Junonia hierta* | 3.31 |  | **0.00** |
|  |  | *Pareronia hippia* | 0.60 |  | 0.55 |
|  |  | *Tajuria cippus* | | 113 | 0.59 |
|  | H1 | *Junonia hierta* | | 45 | 0.35 |
|  |  | *Pareronia hippia* | | 146 | 0.14 |
|  |  | *Tajuria cippus* | | 256 | **0.00** |
|  | H3 | *Junonia hierta* | | 28.5 | 0.56 |
|  |  | *Pareronia hippia* | | 300 | **0.01** |
|  |  | *Tajuria cippus* | | 144 | 0.56 |
|  | S1B | *Junonia hierta* | 3.93 |  | **0.00** |
|  |  | *Pareronia hippia* | 9.56 |  | **0.00** |
|  |  | *Tajuria cippus* | 20.17 |  | **0.00** |
|  | S8 | *Junonia hierta* | 1.95 |  | 0.07 |
|  |  | *Pareronia hippia* | 18.67 |  | **0.00** |
|  |  | *Tajuria cippus* | 14.85 |  | **0.00** |

**Table S5:** Binomial and Wilcoxon rank-sum tests of coefficients of variation (CVs) between males and females for different functional roles of colour patches.

| **Binomial tests** | | | | | | | |
| --- | --- | --- | --- | --- | --- | --- | --- |
| **Colour parameter** | **Comparison** | **Number of trials** | **Number of successes (CV males > CV females)** | |  | | **p-value** |
| **Brightness** | All males vs all females | 163 | 87 | |  | | 0.22 |
|  | Dorsal | 88 | 48 | |  | | 0.23 |
|  | Ventral | 75 | 39 | |  | | 0.41 |
|  | **Aposematic** | | |  | |  |  |
|  | All males vs all females | 38 | 17 | |  | | 0.79 |
|  | Dorsal | 18 | 7 | |  | | 0.88 |
|  | Ventral | 20 | 10 | |  | | 0.59 |
|  | **Mimetic** |  |  | |  | |  |
|  | All males vs all females | 25 | 17 | |  | | 0.05 |
|  | Dorsal | 15 | 10 | |  | | 0.15 |
|  | Ventral | 10 | 7 | |  | | 0.17 |
|  | **Camouflage** | | |  | |  |  |
|  | Ventral | 16 | 6 | |  | | 0.89 |
|  | **Thermoregulatory** | | |  | |  |  |
|  | All males vs all females | 7 | 4 | |  | | 0.5 |
|  | **Sexually selected patches** | | | | |  |  |
|  | Dorsal | 17 | 11 | |  | | 0.17 |
|  | **Non-specific** | | |  | |  |  |
|  | All males vs all females | 72 | 40 | |  | | 0.20 |
|  | Dorsal | 44 | 25 | |  | | 0.23 |
|  | Ventral | 28 | 15 | |  | | 0.42 |
| **Saturation** | All males vs all females | 163 | 79 | |  | | 0.68 |
|  | Dorsal | 88 | 44 | |  | | 0.54 |
|  | Ventral | 75 | 35 | |  | | 0.75 |
|  | **Aposematic** | | |  | |  |  |
|  | All males vs all females | 38 | 17 | |  | | 0.79 |
|  | Dorsal | 18 | 9 | |  | | 0.59 |
|  | Ventral | 20 | 8 | |  | | 0.87 |
|  | **Mimetic** |  |  | |  | |  |
|  | All males vs all females | 25 | 14 | |  | | 0.34 |
|  | Dorsal | 15 | 10 | |  | | 0.15 |
|  | Ventral | 10 | 4 | |  | | 0.83 |
|  | **Camouflage** | | |  | |  |  |
|  | Ventral | 16 | 9 | |  | | 0.4 |
|  | **Thermoregulatory** | | |  | |  |  |
|  | All males vs all females | 25 | 9 | |  | | 0.95 |
|  | **Sexually selected patches** | | | | |  |  |
|  | Dorsal | 17 | 7 | |  | | 0.83 |
|  | **Non-specific** | | |  | |  |  |
|  | All males vs all females | 72 | 38 | |  | | 0.36 |
|  | Dorsal | 44 | 25 | |  | | 0.23 |
|  | Ventral | 28 | 13 | |  | | 0.71 |
| **Hue** | All males vs all females | 163 | 81 | |  | | 0.56 |
|  | Dorsal | 88 | 45 | |  | | 0.46 |
|  | Ventral | 75 | 36 | |  | | 0.68 |
|  | **Aposematic** | | |  | |  |  |
|  | All males vs all females | 38 | 25 | |  | | **0.03** |
|  | Dorsal | 18 | 12 | |  | | 0.12 |
|  | Ventral | 20 | 13 | |  | | 0.13 |
|  | **Mimetic** |  |  | |  | |  |
|  | All males vs all females | 25 | 14 | |  | | 0.34 |
|  | Dorsal | 15 | 8 | |  | | 0.5 |
|  | Ventral | 10 | 6 | |  | | 0.38 |
|  | **Camouflage** | | |  | |  |  |
|  | Ventral | 16 | 7 | |  | | 0.77 |
|  | **Thermoregulatory** | | |  | |  |  |
|  | All males vs all females | 7 | 2 | |  | | 0.94 |
|  | **Sexually selected patches** | | | | |  |  |
|  | Dorsal | 17 | 8 | |  | | 0.68 |
|  | **Non-specific** | | |  | |  |  |
|  | All males vs all females | 72 | 30 | |  | | 0.94 |
|  | Dorsal | 44 | 21 | |  | | 0.67 |
|  | Ventral | 28 | 9 | |  | | 0.98 |

| **Wilcoxon rank-sum tests** | | | | | | |
| --- | --- | --- | --- | --- | --- | --- |
| **Comparison** | **Colour parameter** | **Ecological function** | **Surface** | **W** | **p-value** | **Effect size (r)** |
| **Male-Female** | B1 | Aposematism | Dorsal | 136 | 0.42 | 0.14 |
|  |  |  | Ventral | 211 | 0.78 | 0.05 |
|  |  | Mimicry | Dorsal | 57 | 0.1 | 0.38 |
|  |  |  | Ventral | 51 | 0.36 | 0.23 |
|  |  | Thermoregulatory | Dorsal | 7 | 0.89 | 0.10 |
|  |  |  | Ventral | 4 | 1 | 0.09 |
|  |  | Camouflage | Ventral | 155 | 0.73 | 0.06 |
|  |  | Sexually selected | Dorsal | 132 | 0.39 | 0.16 |
|  |  | Non-specific | Dorsal | 798 | 0.73 | 0.04 |
|  |  |  | Ventral | 358 | 0.72 | 0.05 |
|  | H3 | Aposematism | Dorsal | 164 | 0.96 | 0.01 |
|  |  |  | Ventral | 218 | 0.64 | 0.08 |
|  |  | Mimicry | Dorsal | 37 | 1 | 0.01 |
|  |  |  | Ventral | 44 | 0.76 | 0.08 |
|  |  | Thermoregulatory | Dorsal | 7 | 0.89 | 0.10 |
|  |  |  | Ventral | 4 | 1 | 0.09 |
|  |  | Camouflage | Ventral | 155 | 0.73 | 0.06 |
|  |  | Sexually selected | Dorsal | 103 | 0.77 | 0.06 |
|  |  | Non-specific | Dorsal | 903 | 0.54 | 0.07 |
|  |  |  | Ventral | 291 | 0.4 | 0.12 |
|  | S8 | Aposematism | Dorsal | 120 | 0.19 | 0.22 |
|  |  |  | Ventral | 132 | 0.07 | 0.29 |
|  |  | Mimicry | Dorsal | 48 | 0.39 | 0.20 |
|  |  |  | Ventral | 41 | 0.97 | 0.02 |
|  |  | Thermoregulatory | Dorsal | 4 | 0.34 | 0.41 |
|  |  |  | Ventral | 4 | 1 | 0.09 |
|  |  | Camouflage | Ventral | 146 | 0.97 | 0.01 |
|  |  | Sexually selected | Dorsal | 91 | 0.43 | 0.15 |
|  |  | Non-specific | Dorsal | 942 | 0.33 | 0.11 |
|  |  |  | Ventral | 294 | 0.43 | 0.11 |

**Table S6:** Binomial and Wilcoxon rank-sum tests of coefficients of variation (CVs) between males and females for different colour patches.

| **Binomial tests** | | | | | |  |
| --- | --- | --- | --- | --- | --- | --- |
| **Colour** | **Colour parameter** | **Comparison** | **Number of trials** | **Number of successes (CV males > CV females)** | **p-value** | |
| Black | B1 | All males vs all females | 39 | 19 | 0.63 | |
|  |  | Dorsal | 26 | 13 | 0.58 | |
|  |  | Ventral | 13 | 6 | 0.71 | |
|  | H3 | All males vs all females | 39 | 26 | 0.03 | |
|  |  | Dorsal | 26 | 18 | 0.04 | |
|  |  | Ventral | 13 | 8 | 0.29 | |
|  | S8 | All males vs all females | 39 | 23 | 0.17 | |
|  |  | Dorsal | 26 | 14 | 0.42 | |
|  |  | Ventral | 13 | 9 | 0.13 | |
| Brown | B1 | All males vs all females | 33 | 19 | 0.24 | |
|  |  | Dorsal | 12 | 8 | 0.19 | |
|  |  | Ventral | 21 | 11 | 0.50 | |
|  | H3 | All males vs all females | 33 | 17 | 0.76 | |
|  |  | Dorsal | 12 | 8 | 0.61 | |
|  |  | Ventral | 21 | 9 | 0.81 | |
|  | S8 | All males vs all females | 33 | 15 | 0.50 | |
|  |  | Dorsal | 12 | 6 | 0.19 | |
|  |  | Ventral | 21 | 9 | 0.81 | |
| White | B1 | All males vs all females | 40 | 23 | 0.21 | |
|  |  | Dorsal | 21 | 12 | 0.33 | |
|  |  | Ventral | 19 | 11 | 0.32 | |
|  | H3 | All males vs all females | 40 | 17 | 0.87 | |
|  |  | Dorsal | 21 | 8 | 0.91 | |
|  |  | Ventral | 19 | 9 | 0.68 | |
|  | S8 | All males vs all females | 40 | 16 | 0.92 | |
|  |  | Dorsal | 21 | 10 | 0.67 | |
|  |  | Ventral | 19 | 6 | 0.97 | |
| Yellow | B1 | All males vs all females | 20 | 8 | 0.87 | |
|  |  | Dorsal | 10 | 6 | 0.38 | |
|  |  | Ventral | 10 | 2 | 0.99 | |
|  | H3 | All males vs all females | 20 | 6 | 0.98 | |
|  |  | Dorsal | 10 | 3 | 0.95 | |
|  |  | Ventral | 10 | 3 | 0.95 | |
|  | S8 | All males vs all females | 20 | 6 | 0.98 | |
|  |  | Dorsal | 10 | 3 | 0.95 | |
|  |  | Ventral | 10 | 3 | 0.95 | |
| RedOrange | B1 | All males vs all females | 20 | 9 | 0.75 | |
|  |  | Dorsal | 13 | 5 | 0.87 | |
|  |  | Ventral | 7 | 4 | 0.50 | |
|  | H3 | All males vs all females | 20 | 11 | 0.41 | |
|  |  | Dorsal | 13 | 6 | 0.71 | |
|  |  | Ventral | 7 | 5 | 0.23 | |
|  | S8 | All males vs all females | 20 | 9 | 0.75 | |
|  |  | Dorsal | 13 | 6 | 0.71 | |
|  |  | Ventral | 7 | 3 | 0.77 | |

| **Wilcoxon rank-sum tests** | | | | | | |  |
| --- | --- | --- | --- | --- | --- | --- | --- |
| **Comparison** | **Colour parameter** | **Colour** | **Surface** | **W** | **p-value** | **Effect size (r)** | |
| **Male-Female** | B1 | Black | Dorsal | 310 | 0.62 | 0.07 | |
|  |  |  | Ventral | 88 | 0.88 | 0.04 | |
|  |  | Brown | Dorsal | 83 | 0.55 | 0.13 | |
|  |  |  | Ventral | 262 | 0.31 | 0.16 | |
|  |  | White | Dorsal | 187 | 0.41 | 0.13 | |
|  |  |  | Ventral | 193 | 0.73 | 0.06 | |
|  |  | Yellow | Dorsal | 44 | 0.68 | 0.10 | |
|  |  |  | Ventral | 27 | 0.09 | 0.39 | |
|  |  | Red-Orange | Dorsal | 71 | 0.51 | 0.14 | |
|  |  |  | Ventral | 32 | 0.38 | 0.26 | |
|  | H3 | Black | Dorsal | 433 | 0.08 | 0.24 | |
|  |  |  | Ventral | 112 | 0.17 | 0.28 | |
|  |  | Brown | Dorsal | 83 | 0.55 | 0.13 | |
|  |  |  | Ventral | 209 | 0.78 | 0.37 | |
|  |  | White | Dorsal | 181 | 0.33 | 0.15 | |
|  |  |  | Ventral | 174 | 0.86 | 0.03 | |
|  |  | Yellow | Dorsal | 30 | 0.14 | 0.34 | |
|  |  |  | Ventral | 28 | 0.11 | 0.04 | |
|  |  | Red-Orange | Dorsal | 86 | 0.96 | 0.02 | |
|  |  |  | Ventral | 31 | 0.46 | 0.22 | |
|  | S8 | Black | Dorsal | 398 | 0.28 | 0.15 | |
|  |  |  | Ventral | 98 | 0.51 | 0.14 | |
|  |  | Brown | Dorsal | 88 | 0.38 | 0.19 | |
|  |  |  | Ventral | 181 | 0.33 | 0.15 | |
|  |  | White | Dorsal | 172 | 0.23 | 0.19 | |
|  |  |  | Ventral | 141 | 0.26 | 0.19 | |
|  |  | Yellow | Dorsal | 31 | 0.17 | 0.32 | |
|  |  |  | Ventral | 31 | 0.17 | 0.32 | |
|  |  | Red-Orange | Dorsal | 62 | 0.26 | 0.23 | |
|  |  |  | Ventral | 25 | 1 | 0.02 | |

**Table S7:** Wilcoxon rank-sum tests for coefficients of variation (CVs) between dorsal and ventral surfaces within sex for functional roles and colours.

| **Wilcoxon rank-sum tests** | | | | | | |  |
| --- | --- | --- | --- | --- | --- | --- | --- |
| **Comparison** | **Colour parameter** | **Functional roles** | **Sex** | **W** | **p-value** | **Effect size (r)** | |
| **Dorsal-Ventral** | B1 | Aposematism | Male | 184 | 0.92 | 0.02 | |
|  |  |  | Female | 222 | 0.23 | 0.20 | |
|  |  | Mimicry | Male | 31 | 0.13 | 0.45 | |
|  |  |  | Female | 93 | 0.34 | 0.20 | |
|  |  | Thermoregulatory | Male | 3 | 0.40 | 0.40 | |
|  |  |  | Female | 3 | 0.40 | 0.40 | |
|  |  | Non-specific | Male | 696 | 0.23 | 0.14 | |
|  |  |  | Female | 606 | 0.07 | 0.23 | |
|  | H3 | Aposematism | Male | 149 | 0.38 | 0.15 | |
|  |  |  | Female | 126 | 0.12 | 0.26 | |
|  |  | Mimicry | Male | 26 | 0.44 | 0.24 | |
|  |  |  | Female | 86 | 0.57 | 0.12 | |
|  |  | Thermoregulatory | Male | 8 | 0.63 | 0.27 | |
|  |  |  | Female | 10 | 0.23 | 0.53 | |
|  |  | Non-specific | Male | 494 | 0.24 | 0.14 | |
|  |  |  | Female | 502 | 0.71 | 0.05 | |
|  | S8 | Aposematism | Male | 143 | 0.29 | 0.18 | |
|  |  |  | Female | 145 | 0.32 | 0.17 | |
|  |  | Mimicry | Male | 13 | 0.35 | 0.28 | |
|  |  |  | Female | 64 | 0.57 | 0.12 | |
|  |  | Thermoregulatory | Male | 8 | 0.63 | 0.27 | |
|  |  |  | Female | 5 | 0.86 | 0.13 | |
|  |  | Non-specific | Male | 433 | 0.06 | 0.23 | |
|  |  |  | Female | 439 | 0.62 | 0.06 | |
| **Colour** | | | | | | |  |
| **Dorsal-Ventral** | B1 | Black | Male | 156 | 0.71 | 0.06 | |
|  |  |  | Female | 170 | 0.99 | 0.00 | |
|  |  | Brown | Male | 143 | 0.54 | 0.11 | |
|  |  |  | Female | 134 | 0.78 | 0.05 | |
|  |  | White | Male | 142 | 0.12 | 0.25 | |
|  |  |  | Female | 162 | 0.32 | 0.16 | |
|  |  | Yellow | Male | 66 | 0.25 | 0.27 | |
|  |  |  | Female | 50 | 1.00 | 0.00 | |
|  |  | Red-Orange | Male | 50 | 0.76 | 0.08 | |
|  |  |  | Female | 69 | 0.07 | 0.42 | |
|  | H3 | Black | Male | 193 | 0.49 | 0.11 | |
|  |  |  | Female | 200 | 0.37 | 0.15 | |
|  |  | Brown | Male | 146 | 0.47 | 0.13 | |
|  |  |  | Female | 117 | 0.75 | 0.06 | |
|  |  | White | Male | 160 | 0.29 | 0.17 | |
|  |  |  | Female | 183 | 0.67 | 0.07 | |
|  |  | Yellow | Male | 48 | 0.91 | 0.03 | |
|  |  |  | Female | 47 | 0.85 | 0.05 | |
|  |  | Red-Orange | Male | 49 | 0.82 | 0.06 | |
|  |  |  | Female | 58 | 0.35 | 0.22 | |
|  | S8 | Black | Male | 188 | 0.59 | 0.09 | |
|  |  |  | Female | 167 | 0.96 | 0.01 | |
|  |  | Brown | Male | 179 | 0.05 | 0.35 | |
|  |  |  | Female | 114 | 0.67 | 0.08 | |
|  |  | White | Male | 163 | 0.33 | 0.16 | |
|  |  |  | Female | 196 | 0.94 | 0.01 | |
|  |  | Yellow | Male | 52 | 0.91 | 0.03 | |
|  |  |  | Female | 60 | 0.48 | 0.17 | |
|  |  | Red-Orange | Male | 40 | 0.70 | 0.10 | |
|  |  |  | Female | 55 | 0.49 | 0.17 | |

**Figure S1: Reflectance spectra of the colour patches measured.** UP=Dorsal side, UN=Ventral side, F=Forewing, H=Hindwing. Number of individuals measured is given in parentheses.

**[The next four panels all correspond to Fig. S1]**


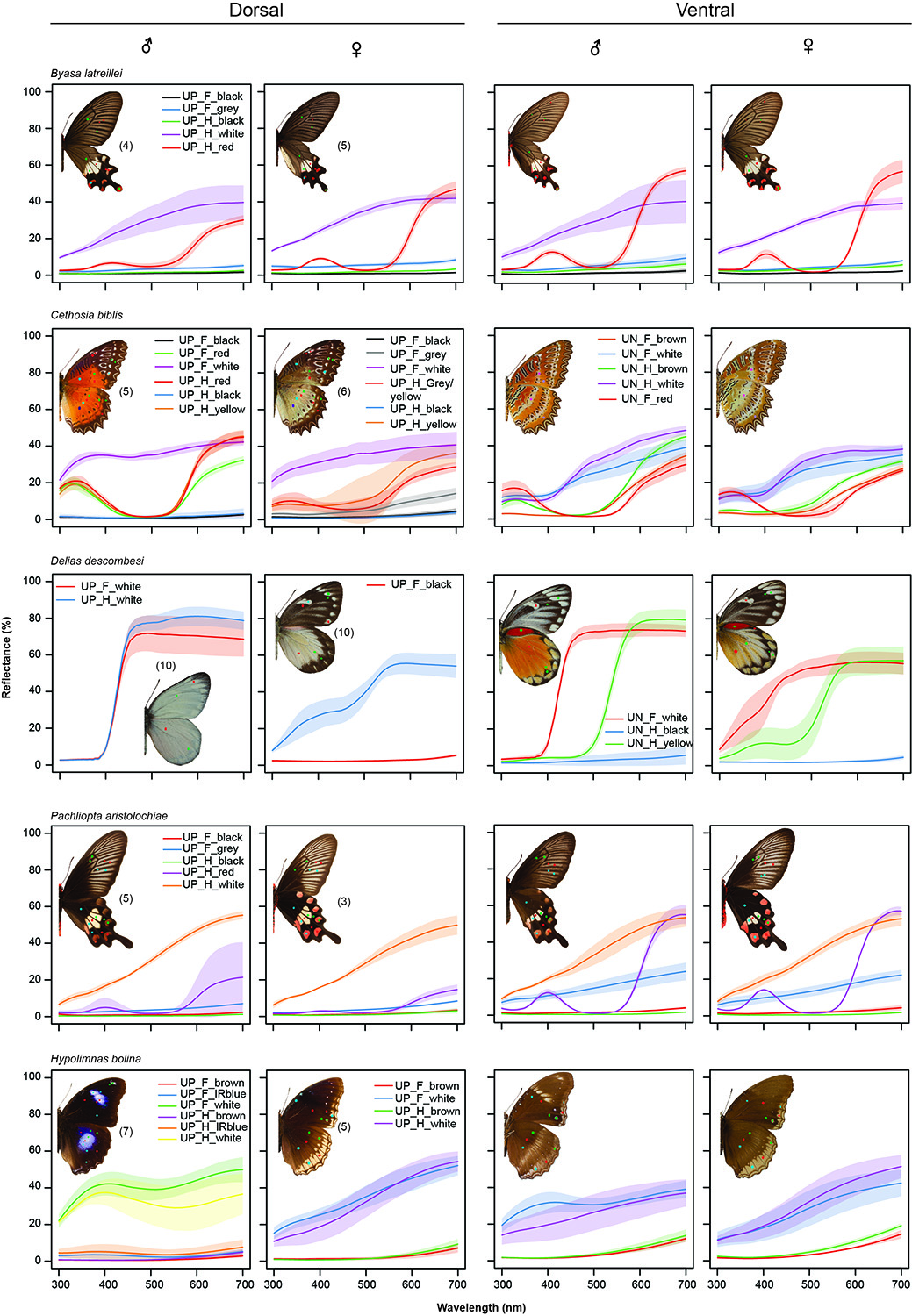


**
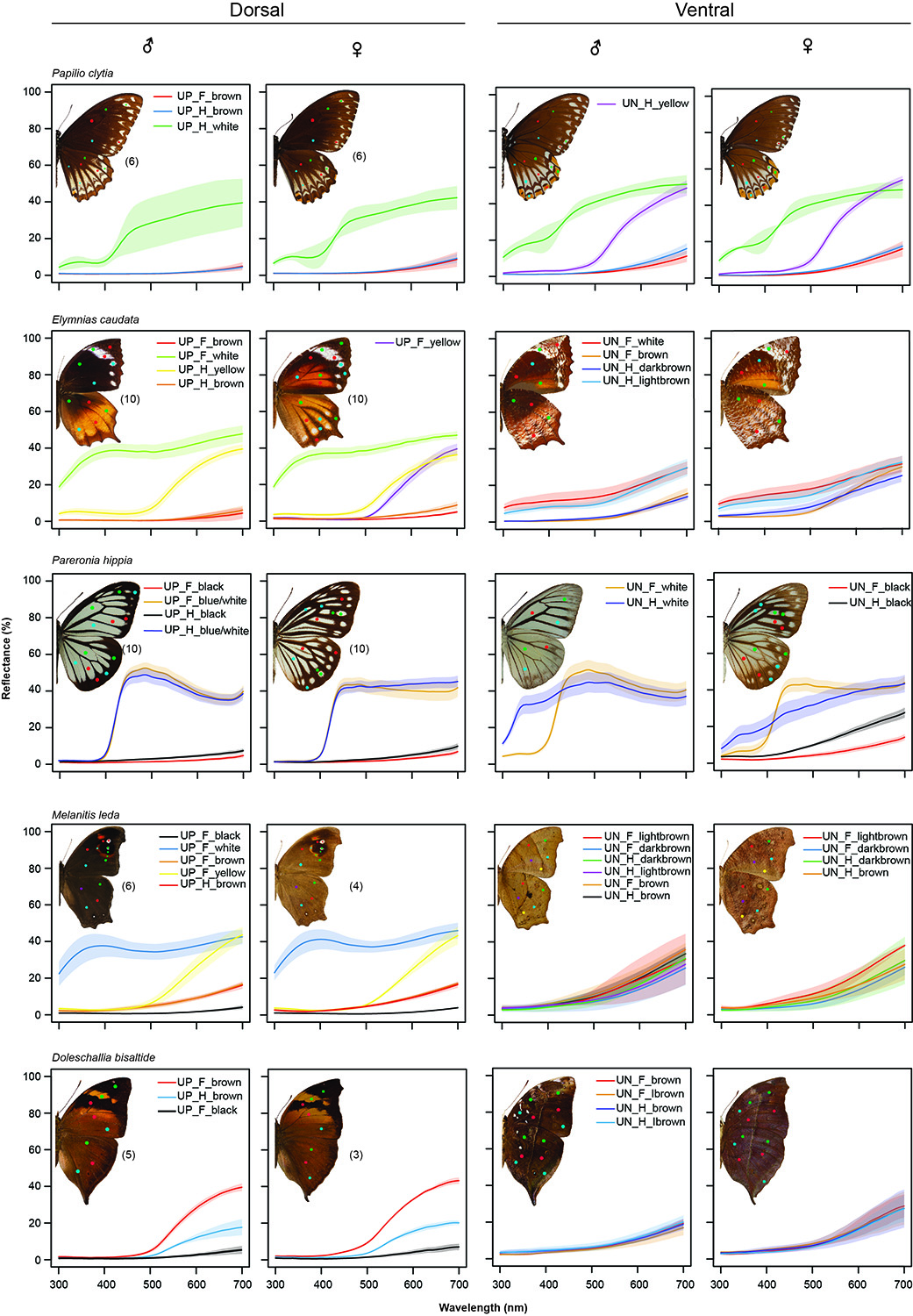
**

**
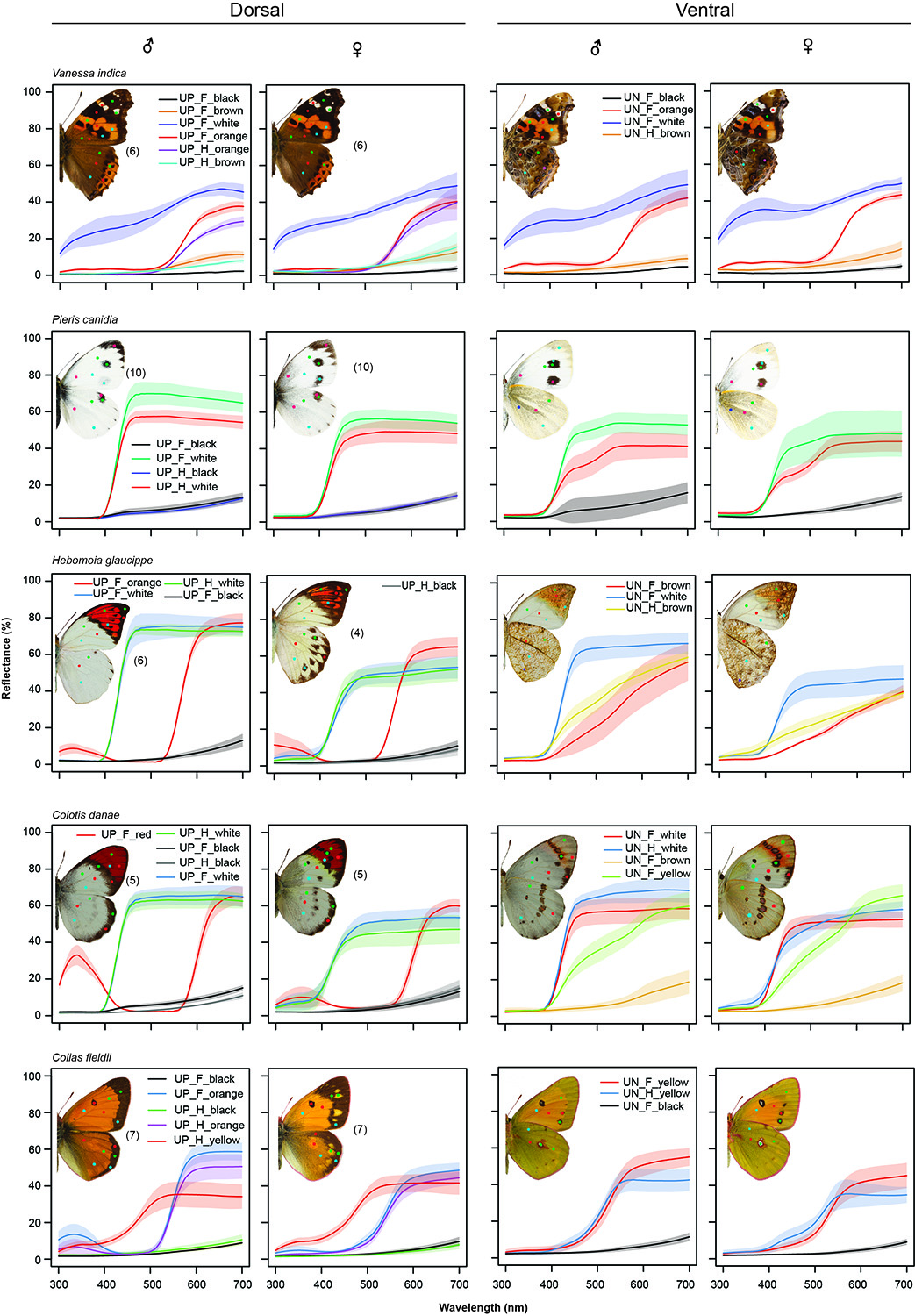
**

**
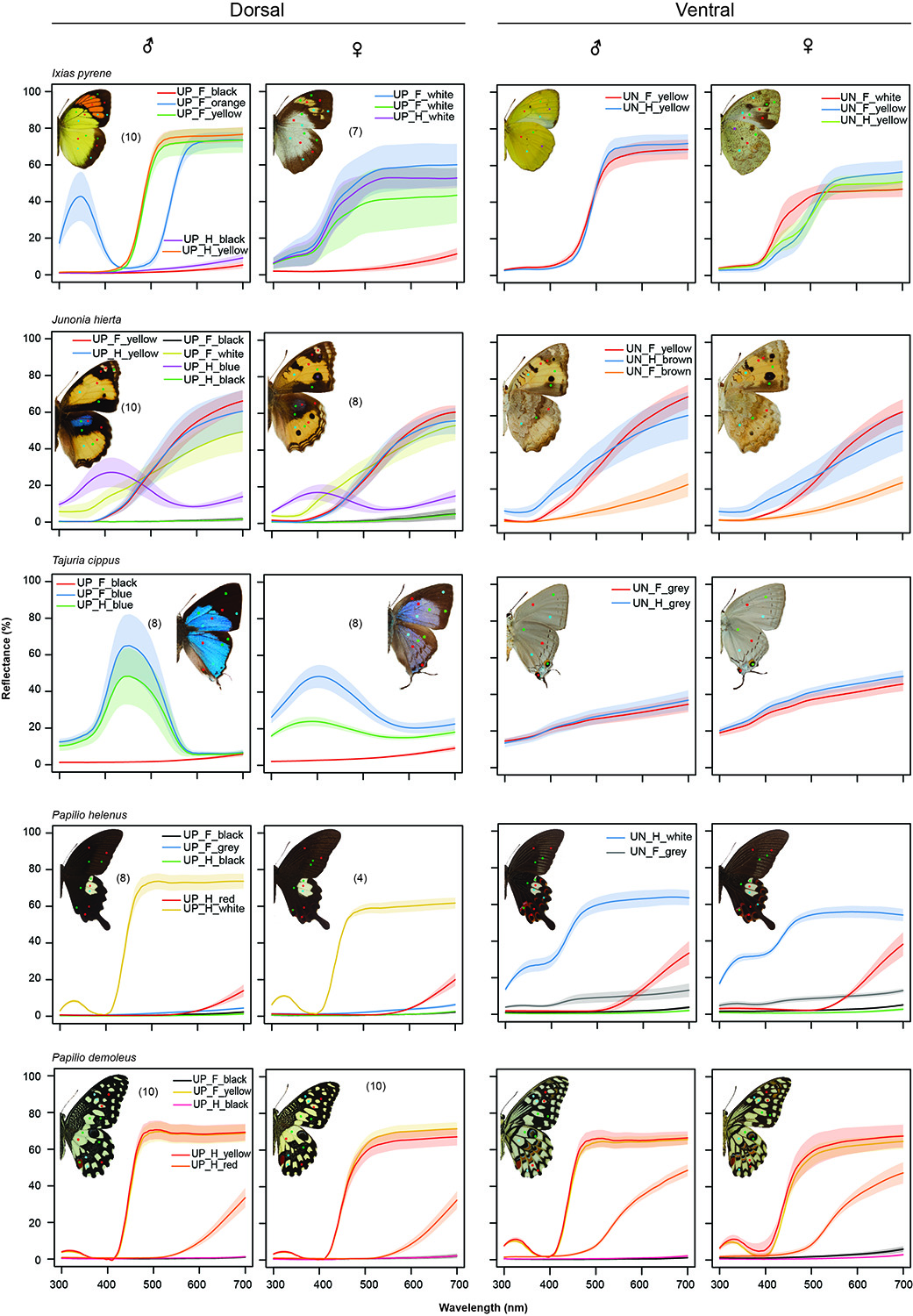
**

**Figure S2:** Boxplots comparing brightness, hue, and saturation between males and females and wing surfaces across colour patches. a: black, b: brown, c: red-orange, d: white, e: yellow, f: blue. The shaded boxes indicate functional roles of colour patches where grey=aposematic patches, pink=mimetic patches, light blue=thermoregulatory patches, brown=cryptic patches, and dark blue=sexually selected patches. Blue patches in (f) are all sexually selected patches. * p<0.05, ** p<0.01, *** p<0.001, **** p<0.0001

**[The next 6 pages all correspond to Fig. S2]**

**
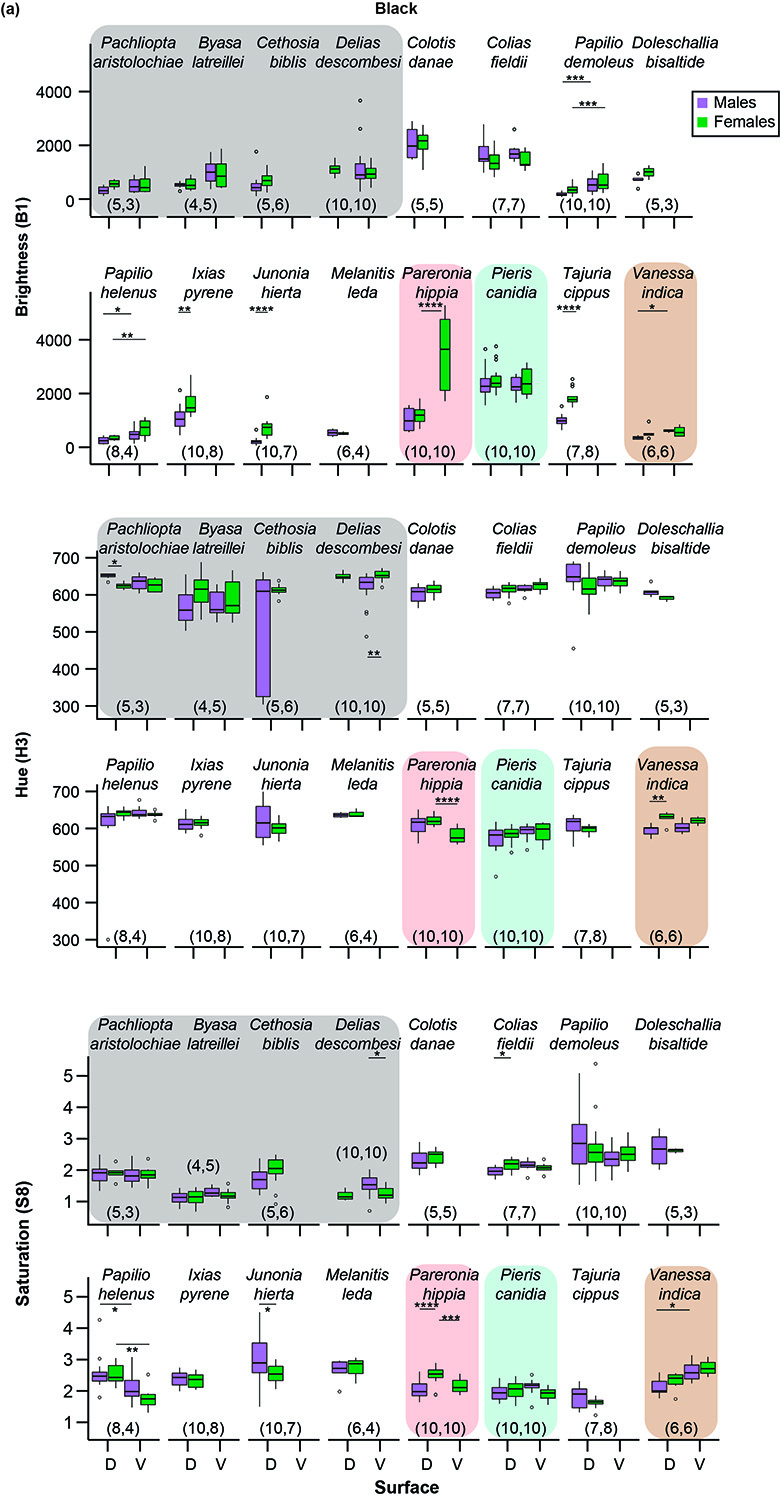
**

**
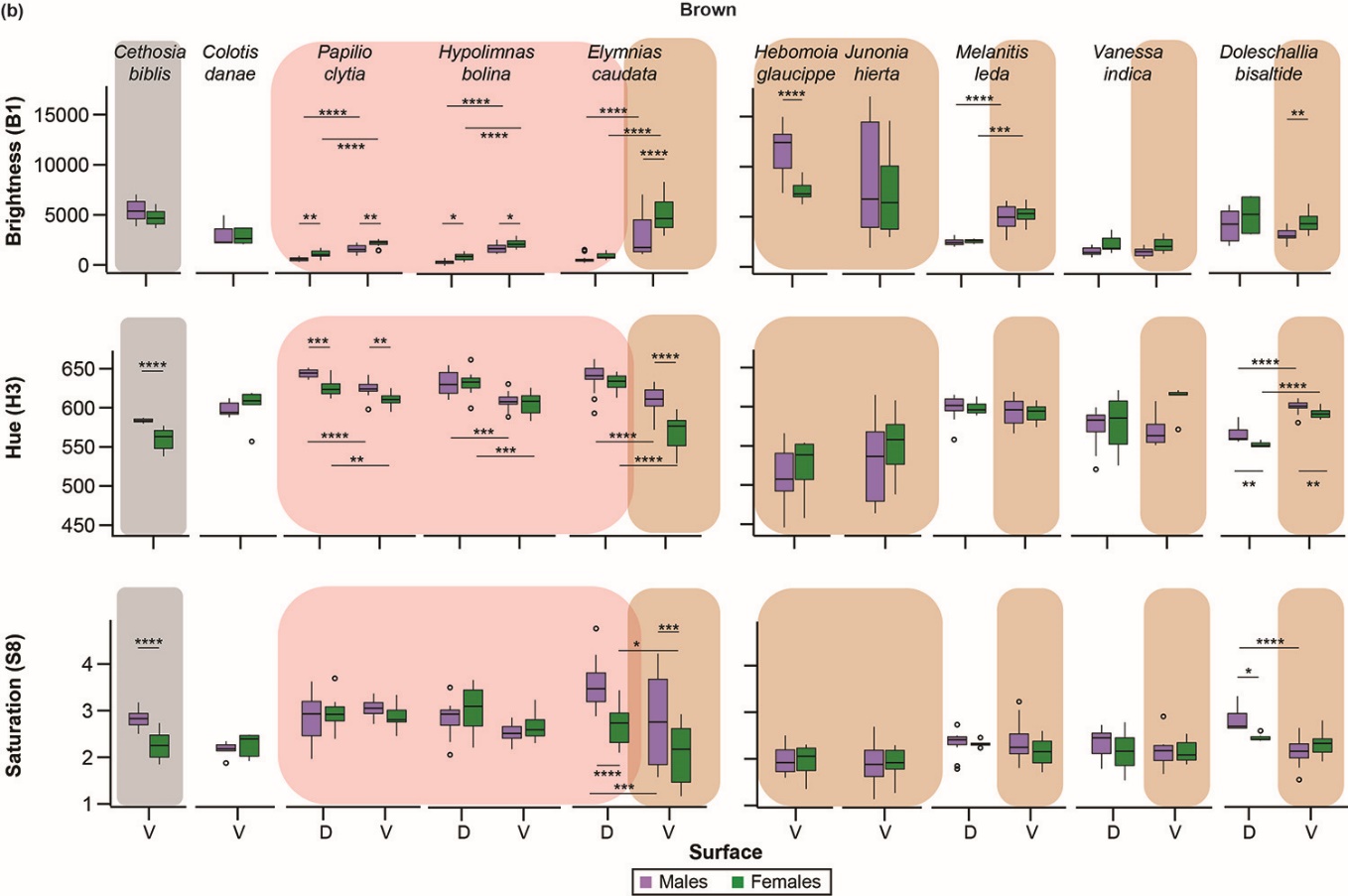
**

**
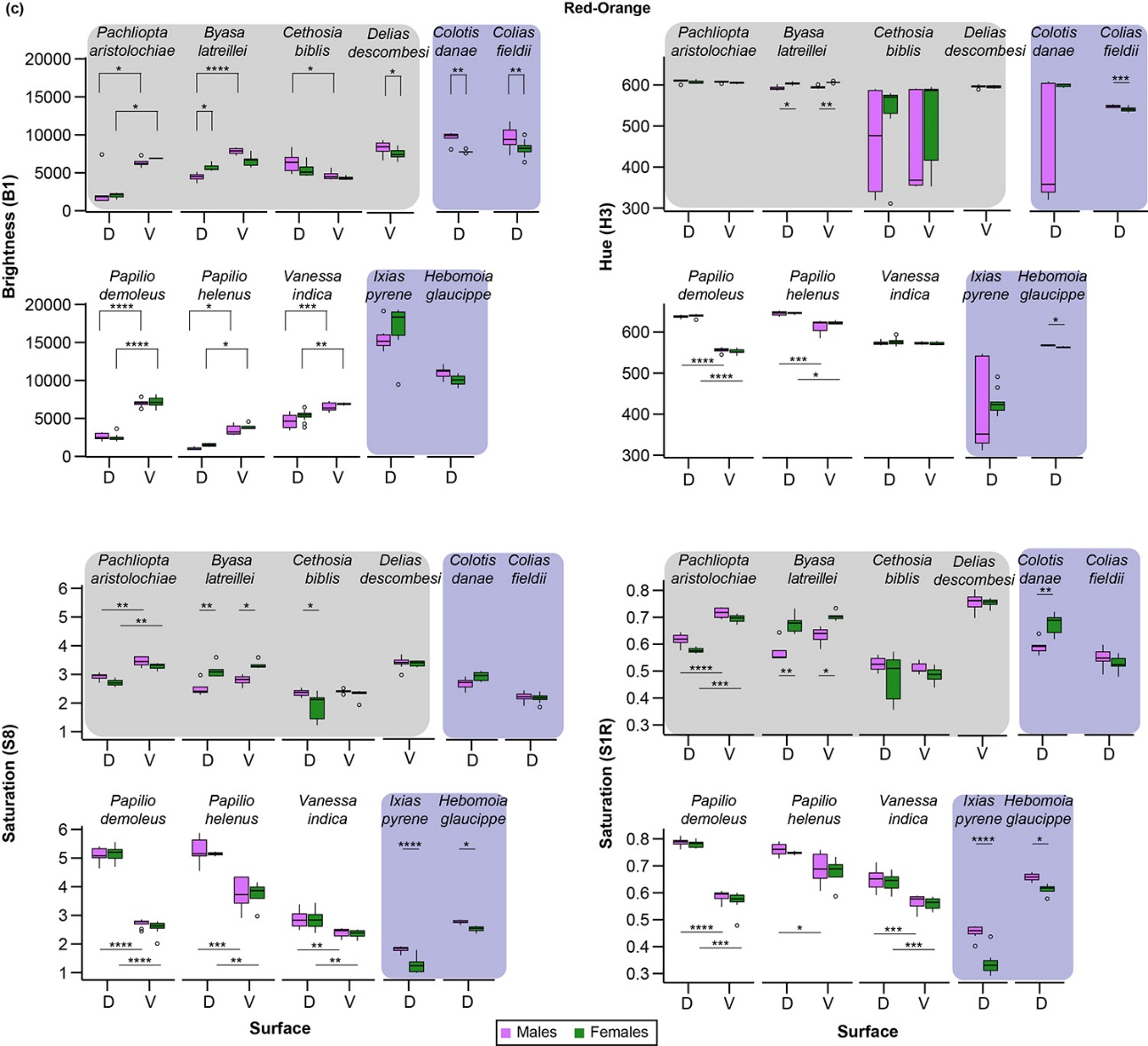
**

**
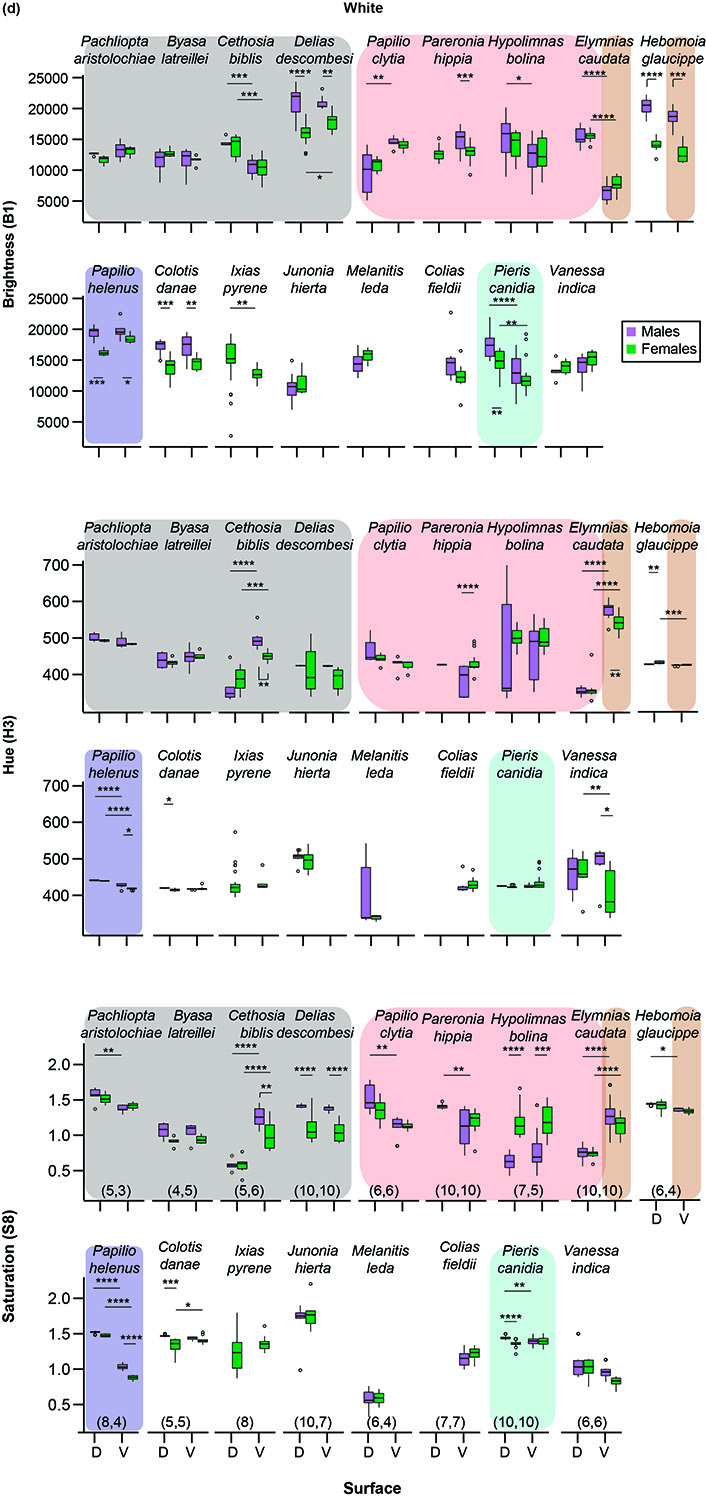
**

**
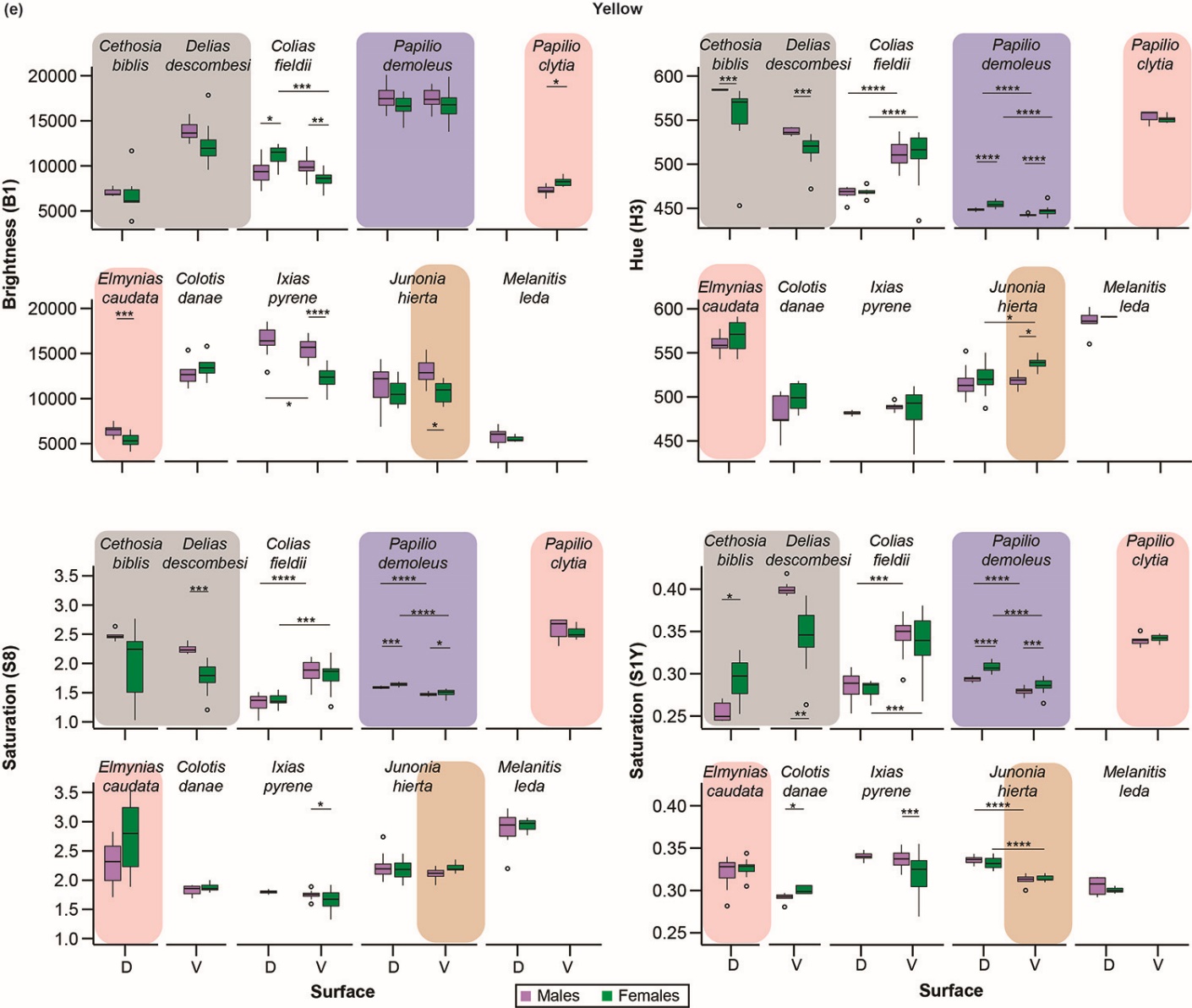
**

**
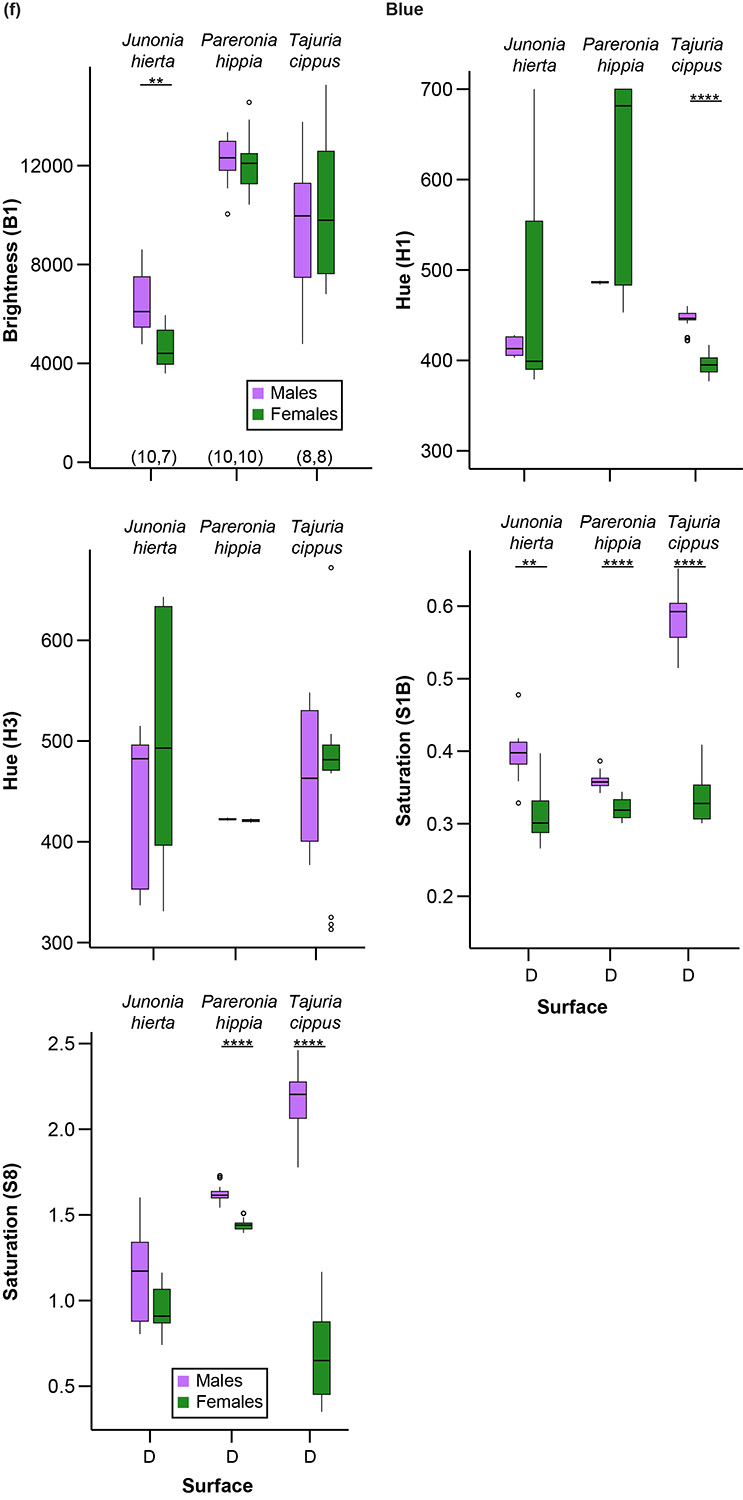
**
